# Comet assay in reconstructed 3D human epidermal skin models—investigation of intra- and inter-laboratory reproducibility with coded chemicals

**DOI:** 10.1093/mutage/get051

**Published:** 2013-10-21

**Authors:** Astrid A. Reus, Kerstin Reisinger, Thomas R. Downs, Gregory J. Carr, Andreas Zeller, Raffaella Corvi, Cyrille A.M. Krul, Stefan Pfuhler

**Affiliations:** ^1^TNO Triskelion, Utrechtseweg 48, 3704 HE Zeist, The Netherlands,; ^2^Henkel AG & Co KGaA, Henkelstrasse67, 40589 Düsseldorf, Germany,; ^3^Procter & Gamble Co., 8700 Mason-Montgomery Road, Mason, OH 45040, USA and; ^4^European Union Reference Laboratory on Alternatives to Animal Testing (EURL ECVAM), IHCP, Joint Research Centre of the European Commission, Via E. Fermi 2749, 21027 Ispra, Italy; ^5^ Present address: F. Hoffmann-La Roche, Ltd, CH-4070 Basel, Switzerland.

## Abstract

Reconstructed 3D human epidermal skin models are being used increasingly for safety testing of chemicals. Based on EpiDerm™ tissues, an assay was developed in which the tissues were topically exposed to test chemicals for 3h followed by cell isolation and assessment of DNA damage using the comet assay. Inter-laboratory reproducibility of the 3D skin comet assay was initially demonstrated using two model genotoxic carcinogens, methyl methane sulfonate (MMS) and 4-nitroquinoline-*n*-oxide, and the results showed good concordance among three different laboratories and with *in vivo* data. In Phase 2 of the project, intra- and inter-laboratory reproducibility was investigated with five coded compounds with different genotoxicity liability tested at three different laboratories. For the genotoxic carcinogens MMS and *N*-ethyl-*N*-nitrosourea, all laboratories reported a dose-related and statistically significant increase (*P* < 0.05) in DNA damage in every experiment. For the genotoxic carcinogen, 2,4-diaminotoluene, the overall result from all laboratories showed a smaller, but significant genotoxic response (*P* < 0.05). For cyclohexanone (CHN) (non-genotoxic *in vitro* and *in vivo*, and non-carcinogenic), an increase compared to the solvent control acetone was observed only in one laboratory. However, the response was not dose related and CHN was judged negative overall, as was *p*-nitrophenol (*p*-NP) (genotoxic *in vitro* but not *in vivo* and non-carcinogenic), which was the only compound showing clear cytotoxic effects. For *p*-NP, significant DNA damage generally occurred only at doses that were substantially cytotoxic (>30% cell loss), and the overall response was comparable in all laboratories despite some differences in doses tested. The results of the collaborative study for the coded compounds were generally reproducible among the laboratories involved and intra-laboratory reproducibility was also good. These data indicate that the comet assay in EpiDerm™ skin models is a promising model for the safety assessment of compounds with a dermal route of exposure.

## Introduction

The standard battery of genotoxicity testing for regulatory purposes normally includes at least two *in vitro* assays to predict the genotoxic potential of pharmaceuticals, industrial chemicals, food additives and ingredients of beauty care products. If a compound shows a positive or equivocal response in at least one *in vitro* assay, additional *in vitro* or *in vivo* genotoxicity assays may be required. *In vitro* assays have the advantage of being relatively inexpensive and allow for rapid screening of compounds. However, the currently used *in vitro* genotoxicity assays for regulatory purposes result in unacceptably high rates of positive outcomes that are often not confirmed *in vivo*. This is especially true for mammalian cell tests such as the chromosomal aberration test, micronucleus test and mouse lymphoma assay (1–4). These so-called ‘misleading’ or ‘false’ positive genotoxins are subjected to additional *in vivo* testing, resulting in increased costs, development delays and unnecessary animal use. This is especially a problem when considering the thousands of chemicals that have to be (re-)evaluated due to the European chemical legislation concerning the Registration, Evaluation, Authorization and Restriction of Chemicals (5). Moreover, compounds in the cosmetics industry cannot be evaluated *in vivo* because animal testing for this purpose is prohibited, as defined in the Seventh Amendment to the Cosmetics Directive (6), which may result in unnecessary loss of new ingredients if genotoxicity assessment relies solely on the current *in vitro* assays (7). For these reasons, improvement of the *in vitro* genotoxicity testing strategy is viewed as high priority.

The skin is the first site of contact for many compounds, including ingredients of beauty and household care products, agrochemicals, dermal pharmaceuticals and industrial chemicals. Currently, for translational reasons, there is a preference towards the development of methods that use human 3D tissue equivalents and a representative route of exposure, rather than methods based on cells cultured in 2D. One of the recommendations made by genotoxicity experts from academia, government and industry during the 5th International Workshop on Genotoxicity Testing (8) and a workshop supported by the European Union Reference Laboratory on Alternatives to Animal Testing (EURL ECVAM) (3) was to investigate the use of new test systems, e.g. reconstructed 3D human epidermal skin models. These models have the advantage of allowing topical application of the compounds, evaluation of formulations and poorly soluble compounds, as well as measurement of local toxicological effects in target cells. Reconstructed 3D human epidermal skin models are considered more relevant than cell lines because of their morphological resemblance to human skin, i.e. the presence of a functional stratum corneum acting as an absorption barrier of compounds and metabolic capacity (9–11). The use of reconstructed 3D human epidermal skin models has increased substantially over the last decade for various toxicological end points. The *in vitro* skin corrosion test was the first test adopted by the OECD, which uses reconstructed 3D human epidermal skin models (OECD TG 431) (12,13) to replace the acute dermal irritation/corrosion test in rats (OECD TG 404), followed a few years later by the *in vitro* skin irritation test in reconstructed 3D epidermal skin models (OECD TG 439) (14,15). Furthermore, reconstructed 3D skin models are used increasingly for investigating the phototoxic (16,17) and sensitization sensitisation potential (18,19) of compounds and these methods have a high potential to be accepted in the near future.

Genetic toxicity is an important toxicological end point as it provides an early prediction of mutagenic and carcinogenic potential. Apart from the low specificity, as mentioned earlier, another shortcoming of the current testing strategy for compounds with a dermal route of exposure is the lack of *in vitro* assays that specifically evaluate genotoxic potential in the skin. Moreover, two of the recommended *in vivo* assays to follow-up positive and equivocal responses from *in vitro* genotoxicity assays, the bone marrow micronucleus test and unscheduled DNA synthesis test using rat hepatocytes, may not be relevant for compounds that are poorly absorbed by the skin. The *in vivo* comet assay, however, is a promising method that can be applied to virtually all types of cells and tissues and an OECD testing guideline is currently being prepared, indicating the great interest of regulatory authorities in this assay (20). The principle of the comet assay is the migration of fragmented DNA in an agarose gel following electrophoresis, resulting in a ‘head’ of intact DNA and a ‘tail’ of fragmented DNA. Single cells are evaluated by fluorescence microscopy after staining with a DNA-binding fluorescent agent, and the % DNA in the tail of the comet is used as a measure for DNA damage (21). Although the *in vivo* comet assay enables evaluation of genotoxic potential in epidermal cells, in the testing strategy, it is positioned as a follow-up *in vivo* assay and it cannot be used for ingredients in beauty care products. Improvement of the current *in vitro* genotoxicity test methods should therefore add support to the use of an *in vitro* testing strategy.

The feasibility of genotoxicity assays in reconstructed 3D human skin models was successfully demonstrated previously (22,23). Cosmetics Europe—The Personal Care Association (formerly known as the COLIPA) and EURL ECVAM supported a global, multi-laboratory project that aimed to develop and validate genotoxicity assays using reconstructed 3D human skin models. The laboratories involved in this project are listed in Table I. Both micronucleus and comet formation were selected as genotoxicity parameters because of their applicability in epidermal cells and coverage of a broad spectrum of DNA damage. Availability of two genotoxicity end points in the same model was preferred to cover different kinds of genetic damage, namely the micronucleus test to detect clastogenicity and aneugenicity, and the comet assay to detect DNA strand breaks, incomplete repair sites and alkali labile sites. The micronucleus test in the EpiDerm™ reconstructed skin model was successfully developed and pre-validated (24–28). The work presented here focuses on the development and pre-validation of the comet assay in the EpiDerm™ model. The comet project team consisted of project leaders and technicians of two cosmetic laboratories [Procter & Gamble (P&G) and Henkel] and an independent research organisation [The Netherlands Organization of Applied Scientific Research (TNO)] (Table I). In Phase 1 of the project, the comet assay methodology using EpiDerm™ was developed and optimised, followed by harmonisation of the methodology across the three laboratories using the model genotoxins, methyl methane sulfonate (MMS) and 4-nitroquinoline-*n*-oxide (4NQO). Phase 2 of the project evaluated the inter- and intra-laboratory reproducibility of the comet assay methodology in EpiDerm™ in the three laboratories using five coded compounds, including MMS, *N*-ethyl-*N*-nitrosourea (ENU) and 2,4-diaminotoluene (2,4-DAT) (genotoxic carcinogens), *p*-nitrophenol (*p*-NP) (genotoxic *in vitro* but not *in vivo* and non-carcinogenic) and cyclohexanone (CHN) (non-genotoxic and non-carcinogenic), selected by a group of independent experts from the areas of genetic toxicology, metabolism and skin carcinogenesis commissioned by the Cosmetics Europe Genotoxicity Task Force (Table I). Results were statistically analysed using predetermined criteria and compared to the *in vivo* genotoxicity and carcinogenicity data for these compounds.

**Table I.** Participants in the Cosmetics Europe 3D reconstructed human skin genotoxicity project (2007–11)

Steering Committee (SC)			
Chair SC	Stefan Pfuhler	The Procter & Gamble company	Cincinnati, OH, USA
Chair Comet assay subteam	Cyrille Krul	TNO	Zeist, The Netherlands
Chair Micronucleus assay subteam 2007–10 Since 2011	Marylin Aardema Rodger Curren	The Procter & Gamble company Institute for In Vitro Sciences	Cincinnati, OH, USA Gaithersburg, MD, USA
Members	Kerstin Reisinger	Henkel AG & Co KgaA	Düsseldorf, Germany
	Gladys Ouédraogo-Arras	L’Oréal Life Sciences Research	Aulnay sous bois, France
Cosmetics Europe Project Management	Monique Fairley (2007–10) João Barroso (since 2011)	Cosmetics Europe	Brussels, Belgium
Representative from EURL ECVAM	Raffaella Corvi	European Union Reference Laboratory on Alternatives to Animal Testing	Ispra, Italy
Chemical Selection Committee			
Chemical Selection Expert Team	David Kirkland	Covance Laboratories	Harrogate, UK
	Thomas Slaga	UTHSCSA-Pharmacology	San Antonio, TX, USA
	Johannes Doehmer	GenPharmTox BioTech AG	Planegg/Martinsried, Germany
	Günther Speit	Universität Ulm	Ulm, Germany
Laboratories involed in the 3D skin comet assay project			
The Procter & Gamble company	Stefan Pfuhler, Andreas Zeller, Thomas Downs, Laurence Richoz, Linda Corbino, Jessica Jester		Cincinnati, OH, USA
Henkel	Kerstin Reisinger, Norbert Mendorf		Düsseldorf, Germany
TNO	Cyrille Krul, Astrid Reus, Michèle van den Wijngaard, Mustafa Usta		Zeist, The Netherlands

## Materials and methods

### Compounds, reagents and media

New maintenance medium (NMM) (EPI-100-NMM) was obtained from MatTek Corporation, Ashland, MA, USA. All test compounds (Table II) were purchased from Sigma–Aldrich. Covance Laboratories coded the test compounds for the Phase 2 studies and shipped them to the participating laboratories. Seaplaque™ GTG™ low melting agarose was obtained from Lonza, Basel, Switzerland. SYBR Gold was obtained from Invitrogen, Life Technologies Corporation, Carlsbad, CA, USA. Other reagents and media were obtained from well-known suppliers but were not harmonised between the laboratories because differences in suppliers of these materials were not considered to negatively affect the quality of the studies.

**Table II.** List of compounds tested

Compound	CAS number	Carcinogenicity and genotoxicity data	Expected response in EpiDerm™ comet assay
MMS	66-27-3	Carcinogenic *in vivo*, positive in *in vitro* and *in vivo* genotoxicity assays (reviewed in ref. 29)	+
4NQO	56-57-5	Carcinogenic *in vivo*, positive in *in vitro* and *in vivo* genotoxicity assays (30–32)	+
ENU	759-73-9	Carcinogenic *in vivo*, positive in *in vitro* and *in vivo* genotoxicity assays (reviewed in ref. 29)	+
2,4-DAT	95-80-7	Carcinogenic *in vivo*, positive in *in vitro* and *in vivo* genotoxicity assays (reviewed in ref. 29)	+
*p*-NP	100-02-7	Non-carcinogenic, positive in *in vitro* genotoxicity assays (reviewed in ref. 29)	-
CHN	108-94-1	Non-carcinogenic, non-genotoxic in *in vitro* assays (reviewed in ref. 29)	-

### Skin models and treatment

EpiDerm™ EPI-200-MNA skin models were obtained from MatTek Corporation. EpiDerm™ consists of normal human epidermal keratinocytes obtained from neonatal foreskin that have been cultured to produce a stratified, highly differentiated, organotypic tissue model of the human epidermis. EpiDerm™ tissue consists of metabolically and mitotically active cells, which are organised into basal, spinous and granular layers along a multilayered stratum corneum and has an air–liquid interface that allows test materials to be directly applied to the surface of the tissue (adopted from MatTek website). Upon arrival at the laboratories, skin models were acclimatised overnight in 6-well plates containing 1 ml NMM in a humidified incubator at 37°C and 5% CO_2_. On the day of treatment, skin models were placed in 1 ml fresh NMM. Test compounds were dissolved in acetone at a maximum concentration of 100mg/ml (10% solution) or lower if limited by solubility and/or cytotoxicity, followed by the preparation of serial dilutions with maximal 3.16-fold spacing between doses.

Just before treatment, any moisture on the skin surface was removed with a cotton tip, followed by topical application of 10 μl of the dose solutions directly on the surface of the EpiDerm™ skin model (equivalent to 16 μl/cm^2^ skin). Concurrent untreated and solvent controls were run in each assay. In experiments with coded compounds, 5 or 16 μg/cm^2^ MMS was used as a positive control. During the 3-h treatment period, skin models were placed in a humidified incubator at 37°C and 5% CO_2_. Four skin models per dose group and at least three dose levels per test compound were used, unless otherwise stated. Each compound was tested in at least two independent experiments in each lab. Cytotoxicity was measured simultaneously using trypan blue dye exclusion (Henkel and TNO), ethidium bromide/acridine orange staining (P&G) and/or relative total cell counts.

### Isolation of epidermal cells

The cell isolation procedure was based on the methods described by Curren *et al.* (22) with minor modifications. All steps were performed at room temperature, unless otherwise mentioned. After the 3-h treatment period, skin models were submerged in 5 ml phosphate-buffered saline (PBS) for 5–15min in a 12-well plate, then in 5 ml PBS containing 0.1% EDTA for another 5–15min, followed by incubation in a 0.25% trypsin, 0.02% EDTA solution for 15min (1 ml in the well and 0.5 ml on top of the skin membrane), the latter being pre-warmed to 37°C before use. Thereafter, the skin model was removed from the insert with forceps and placed in the same well as the insert, and 1 ml of cold Dulbecco’s modified Eagle’s medium or minimum essential medium Eagle supplemented with 10% foetal calf serum was added to the well and to the insert. The medium was pipetted up and down seven to eight times to obtain single cells in suspension. After filtration through a 40-μm cell strainer and centrifugation (200 × g, 5min), the supernatant was removed except for ~50 µl in which the cells were resuspended, followed by the addition of 0.5–1.0 ml 0.5% low melting agarose in PBS (pre-warmed to 37°C, the volume was dependent on the size of the cell pellet), and the preparation of three comet slides per skin model.

### Comet assay

The comet assay procedure was based on the method described by Singh *et al.* (33) and others (20,21). Briefly, 75 μl of the cell suspension in low melting agarose was added to a microscope slide, which was pre-coated with 1.0% normal melting agarose in PBS and allowed to dry. The cell suspension was covered with a glass cover slip to spread it evenly over the slide and the slides were placed at 4°C for 3–5min. Following coagulation of the low melting agarose and removal of the cover slip, slides were incubated overnight in lysis buffer (2.5M NaCl, 0.1M Na_2_EDTA, 0.01M Tris and 1% Triton X-100 in distilled water, pH 10) at 4°C. Thereafter, slides were incubated for 20min in ice-cold electrophoresis buffer (0.3M NaOH and 0.001M Na_2_EDTA in distilled water, pH > 13) for DNA unwinding, followed by 30-min electrophoresis in a Roth electrophoresis chamber (catalogue number N610.1, Carl Roth GmbH & Co. KG, Karlsruhe, Germany) filled with ice-cold electrophoresis buffer (1V/cm, 450 ± 50 mA), a 5-min incubation in neutralisation buffer (0.4M Tris in distilled water, pH 7.5) and a 5-min dehydration in >96% ethanol.

### Slide scoring

**Fig. 1.**
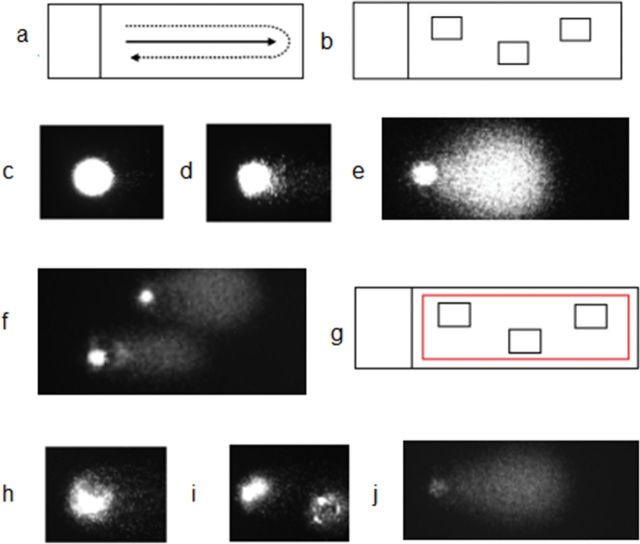
Overview of the standard operating procedure for slide analysis. Even if a slide shows a homogenous distribution of cells, do not concentrate on one region but move across the slide along the arrow-defined manner (follow the dotted arrow in case there are not enough cells in one horizontal movement) (**a**), if many cells are present on the slide, randomly select three to four regions to represent the whole slide and (**b**), three examples of cells that fulfil the criteria of a round-shaped head, which is intense and homogenously stained (**c**–**e**). Overlapping cells (**f**), cells outside the red frame that are too close to the border of the slide (**g**), cells with an irregular shaped, leaky head (**h** and **i**) or ghost cells (**j**) should not be analysed.

Slides were coded to prevent operator bias during analysis of the slides. Just before analysis, slides were stained with SYBR gold [diluted 10 000× in a Tris–EDTA buffer (10mM Tris and 1mM Na_2_EDTA in distilled water, pH 7.5)]. At least two slides per skin model were analysed. Fifty nuclei per slide were randomly measured with Comet Assay IV software (Perceptive Instruments, Suffolk, UK). Even if a slide showed a homogenous picture, the slide was moved from left to right instead of concentrating on regions (Figure 1a), and if many cells were present on the slide, three to four regions were randomly selected to represent the whole slide (Figure 1b). Every cell with a round-shaped head, which was intense and homogenously stained, was measured (see Figure 1c–e). Overlapping cells (Figure 1f), cells close to the border of the slide (Figure 1g) and cells that did not meet the above-mentioned criteria were excluded from analysis (e.g. cells with a leaky head, see Figure 1h and i). Ghost cells, with a small head and a diffuse and large tail (Figure 1j), were excluded from analysis, but their presence was counted as an indication of cytotoxicity if it fulfilled the criteria for a real comet (no overlapping, no border effects, etc.). Slides were discarded when <50 analysable cells were found (e.g. when there were only ghost cells present on the slide or when the total number of cells on the slide was too low).

### Statistics

Prior to all data processing, a variance-stabilising transformation was applied to the % tail DNA values at the cell level. Percentages were converted to proportions, *p*, and then the transformed response was calculated as: sin^−^^1^√*p*. While this variance-stabilising transformation is derived for binomial proportion data, it also proves useful for comet data (34). Slide-level data for 50 nuclei were subsequently summarised by a median value, in order to limit the influence of large % tail DNA values that skew the observed distributions of % tail DNA within a slide, particularly in control responses. Slide-level median values were then averaged into a single summary measure of % tail DNA for each tissue tested, on the transformed response scale described above. Because treatments were applied to tissues, the tissue is the experimental/statistical unit, and all statistical analyses of treatment effect were performed on these tissue-level summaries. Data at each of these levels of summarisation (cell/slide/tissue) were summarised numerically and graphically to inspect for the possibility of outliers and to assess the quality of data obtained.

Because treatment-related decreases in the % tail DNA are not relevant to this assay, statistical analyses are done in a one-sided manner for an increase in response due to treatment. Two types of statistical tests are performed on each experiment. One is the pairwise comparison of each treatment to its control, performed by the method of Dunnett (35). The second is a test for dose–response trend based upon simple linear regression. The trend test is of secondary importance to the evaluation of results, being most useful for alerting investigators to the possibility of a slight treatment effect in the absence of any one dose level being *P* < 0.05 in the pairwise comparisons. Any positive control groups were analysed separately from the evaluation of the test material.

### Evaluation criteria

The assay was considered valid if the positive control compound caused a statistically significant increase in group mean % tail DNA compared to the solvent control and if the arithmetic mean % tail DNA of the untreated and solvent controls was ≤30%. The 30% cut-off value for the background was derived from the upper limit of the 90% confidence interval of all experiments performed during Phases 1 and 2 of the project (>20 experiments). Exceeding this value will usually result in increased variability of the data. A dose group was considered valid if included at least three evaluable tissues.

A test compound was considered positive for genotoxicity if it had at least one study with two or more (consecutive) dose levels producing statistically significant increases in % tail DNA, or if the highest concentration produced a statistically significant increase in % tail DNA in the absence of a relevant cytotoxic effect (>30%) and a significant effect was reproduced in an independent study. If a test compound induced a significant increase only at a dose other than the highest dose, it was considered positive only if the trend test was positive, and it was reproducible.

A test compound that did not demonstrate a relevant increase of the % tail DNA was considered non-genotoxic.

## Results and discussion

### Development of the EpiDerm™ comet assay methodology and transferability to other laboratories

The enzymatic cell dissociation procedure was based on the method described for the 3D skin micronucleus assay (22) and optimised for the comet assay to obtain sufficient single numbers of cells with acceptable % tail DNA values in untreated and solvent controls. The method was then successfully transferred from P&G to TNO and Henkel, as demonstrated by a sensitive detection of two model genotoxins (MMS and 4NQO, see Reproducibility). As a next step, the comet assay protocols from the different laboratories were harmonised to enable appropriate comparison of results by using identical protocols, including the use of the same model of the electrophoresis chamber, and harmonised electrophoresis conditions across all laboratories. Finally, a standard operating procedure for slide analysis was designed (Figure 1).

#### Solvents. 

Several solvents were investigated for their applicability in the comet assay using reconstructed 3D human skin models and as a result, acetone and ethanol were considered suitable solvents. In addition to having no effect on background % tail DNA values, acetone was the preferred solvent because it facilitates absorption of compounds in the epidermis and evaporates quickly from the tissue surface. This solvent was selected because it also had low background in micronucleus assays in EpiDerm™ compared to other solvents (22,24–28). The % tail DNA values in tissues treated with 70% ethanol were comparable with those treated with acetone (group mean ± SD for acetone vs. 70% ethanol: 22.5±9.9% vs. 20.0±3.5%, one experiment with *n* = 4 tissues/group at TNO), as did tissues treated with 90% ethanol (acetone vs. 90% ethanol: 31.9±14.0% vs. 29.8±6.8%, one experiment with *n* = 4 tissues/group at TNO) and 100% ethanol (acetone vs. 100% ethanol: 29.7±12.7% vs. 28.6±5.4%, mean ± SD of *n* = 8 experiments at P&G), indicating that the solvents other than acetone can be used. Tissues treated with 70% ethanol also exhibited similar % tail DNA values to acetone in the *ex vivo* skin comet assay, as did 100% ethanol and 50% ethanol (36). Selection of suitable solvents is very important because the solvent may influence skin absorption and subsequent bioavailability (37,38) and we wanted to avoid solvents that decreased absorption and thus the sensitivity of the assay. Moreover, solvents may also induce adverse effects, e.g. decreased proliferation compared to untreated EpiDerm™ models was reported for dimethyl sulfoxide (DMSO) and saline (22,26) in micronucleus assays. In the 3D skin comet assay, PBS and DMSO were also considered not suitable for the comet assay because they resulted in % tail DNA values >30% (data not shown), most likely due to the induction of a stress response as a result of reduced oxygen supply since these solvents do not readily penetrate and remain on surface of the skin, but this was not investigated further in the present study. The 30% cut-off as maximum value for the % tail DNA of the solvent or negative control is considered necessary in order to maintain a high enough dynamic range of the assay, i.e. to protect its sensitivity to detect increases in DNA damage. A similar cut-off value (25%) was applied in a study using *ex vivo* human skin tissues (36). The % tail DNA values of untreated or solvent control skin tissues are generally higher compared to cell cultures, which might be related to the isolation procedure since a more invasive enzyme treatment is necessary to obtain single cells from the compact skin matrix when compared to cell cultures.

### Reproducibility

#### Phase 1: intra-laboratory reproducibility—testing of two model compounds—MMS and 4NQO. 

**Fig. 2.**
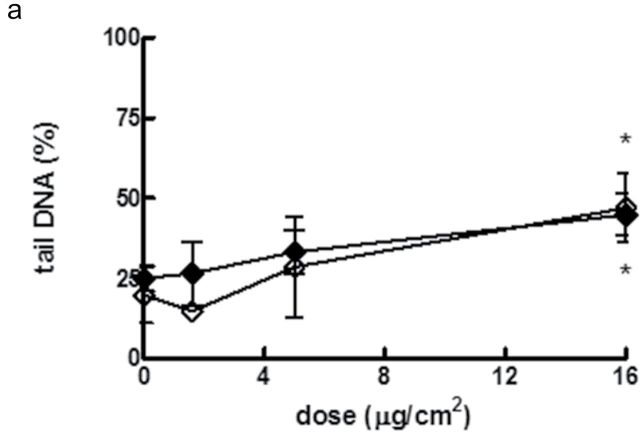

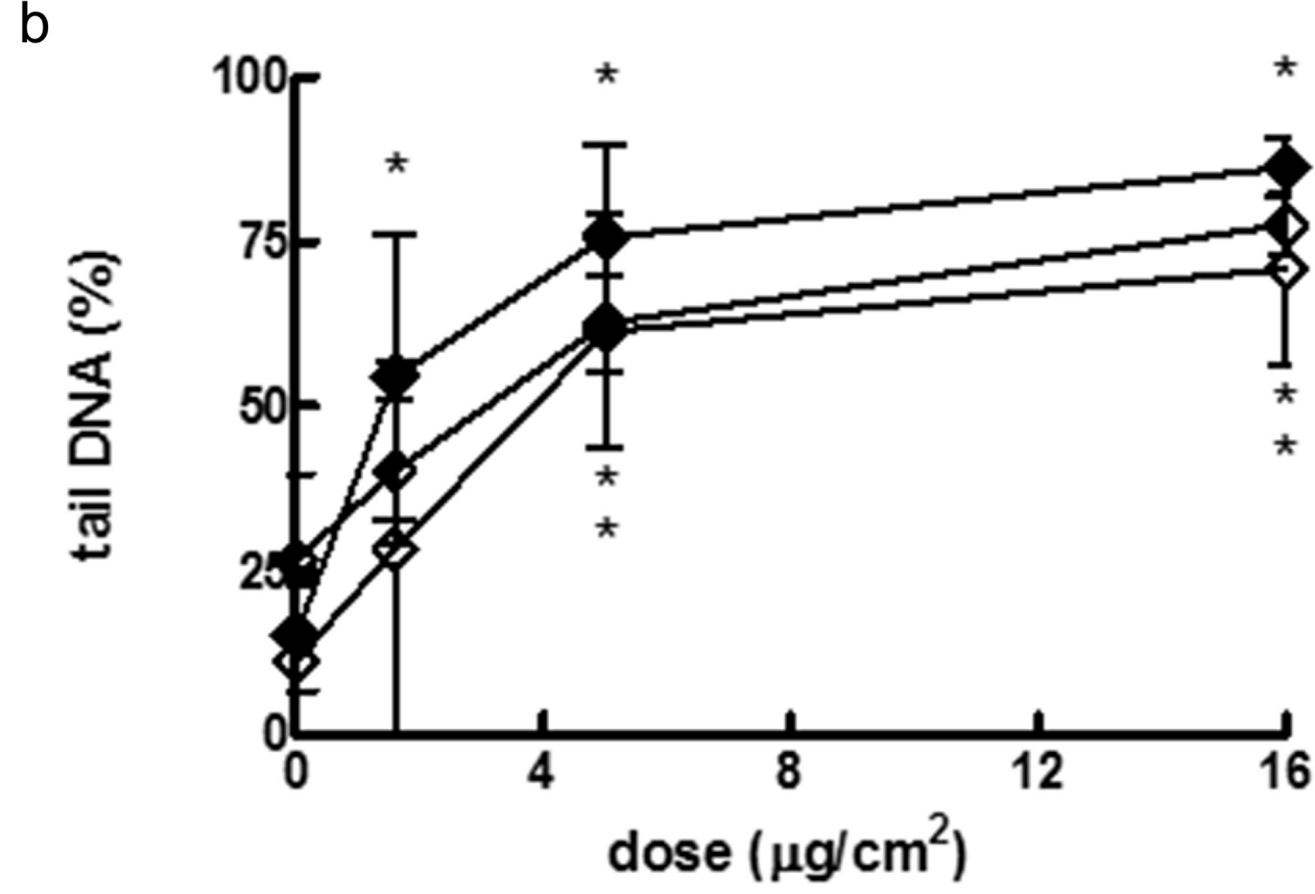

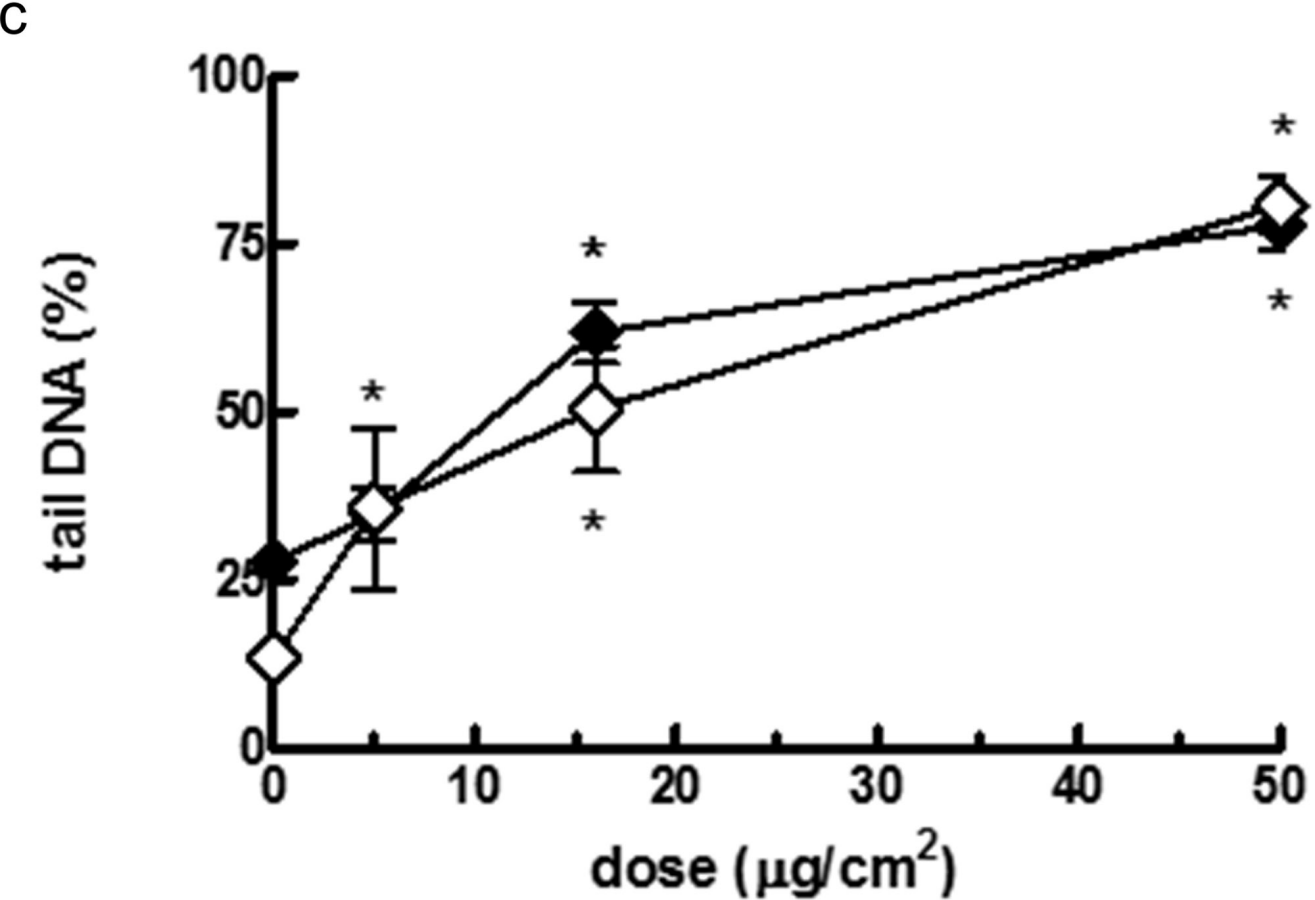
Percentage tail DNA in epidermal cells from EpiDerm™ after 3-h treatment with MMS at (**a**) Henkel, (**b**) P&G and (**c**) TNO. Within each set of results, separate studies are represented with different symbol shapes. Values are mean ± SD. *Significant increase over concurrent solvent control (*P* < 0.05).

**Fig. 3.**
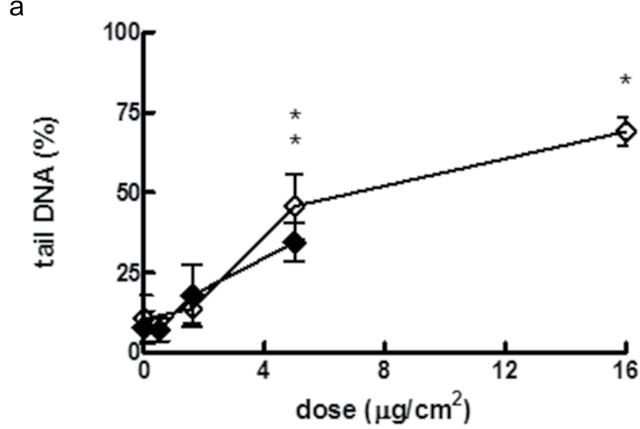

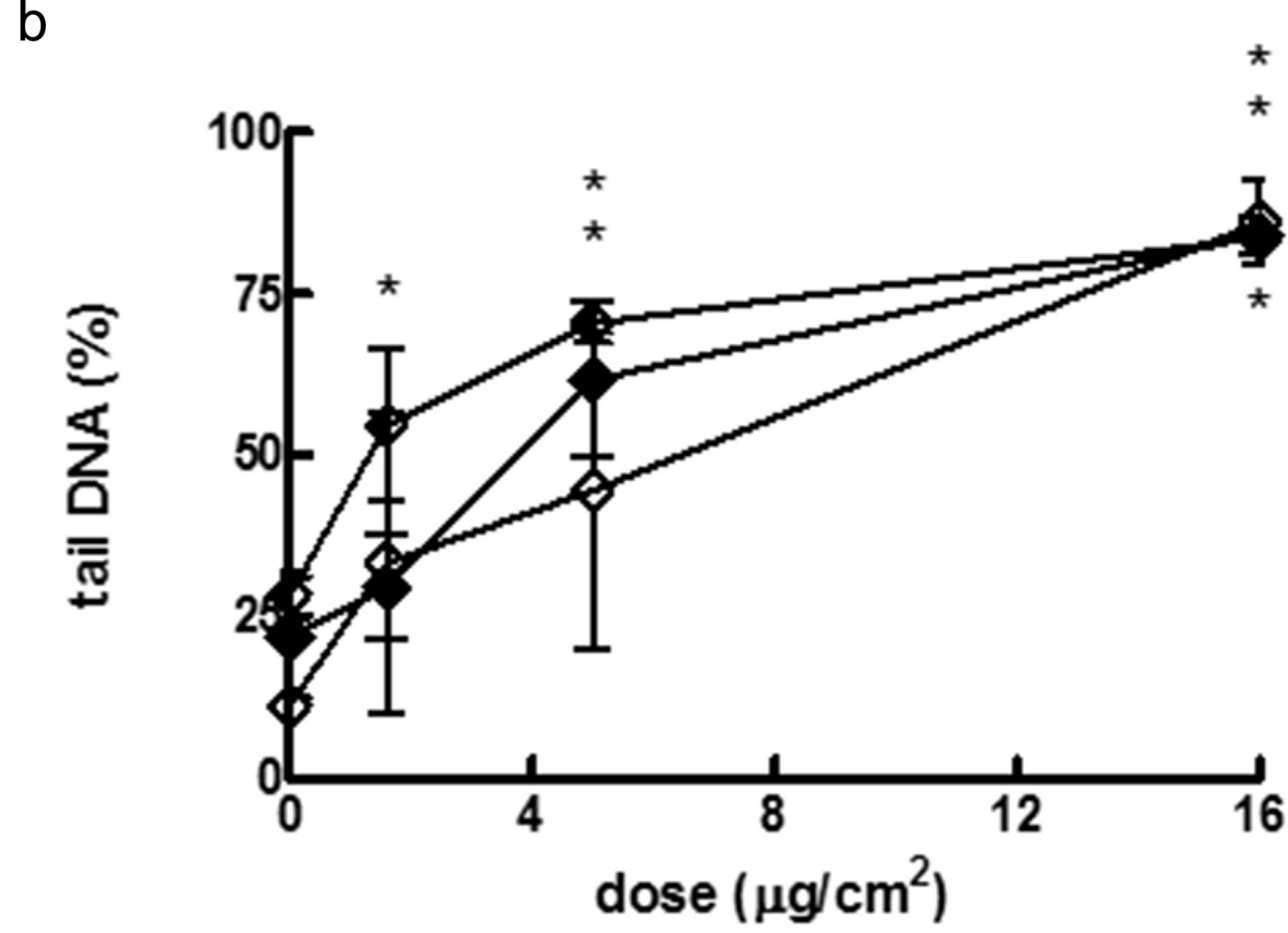

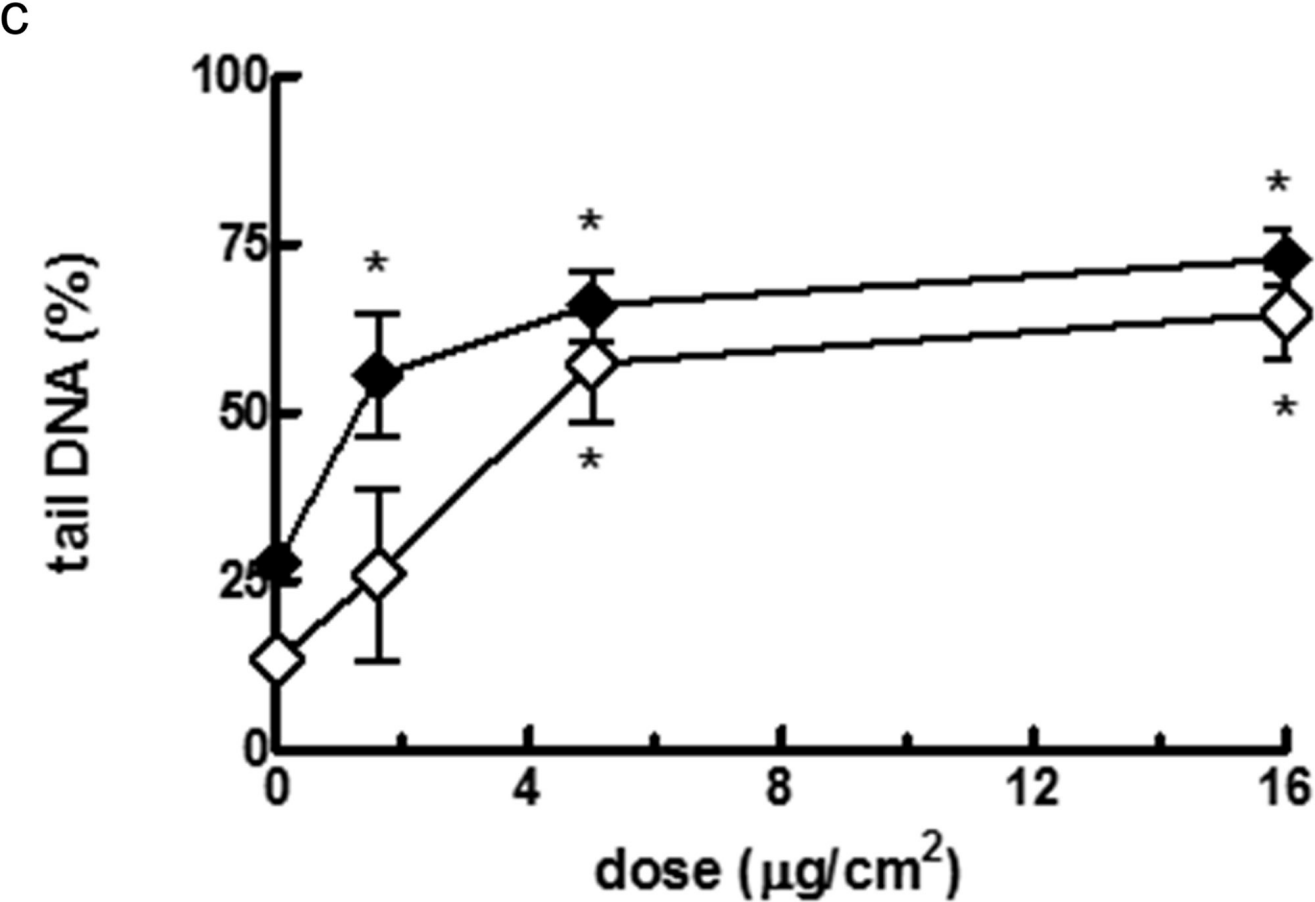
Percentage tail DNA in epidermal cells from EpiDerm™ after 3-h treatment with 4NQO at (**a**) Henkel, (**b**) P&G and (**c**) TNO. Within each set of results, separate studies are represented with different symbol shapes. Values are mean ± SD. *Significant increase over concurrent solvent control (*P* < 0.05).

The model genotoxins, MMS and 4NQO, resulted in dose-related and statistically significant increases in % tail DNA, which were reproducible in every experiment within and across the three laboratories (Figures 2 and 3). These data confirm the positive findings with MMS and 4NQO in skin observed by others in the micronucleus assay (27,39,40). As previously reported for *ex vivo* human skin tissues by Reus *et al.* (36), MMS was also considered a suitable positive control for the comet assay in EpiDerm™.

#### Phase 2: inter-laboratory reproducibility of the EpiDerm™ comet assay methodology—testing of five coded chemicals. 

P&G, Henkel and TNO each tested five compounds that were coded using different codes for each laboratory and each dissolved in acetone (the solvent used by each laboratory). These compounds were MMS, ENU, 2,4-DAT (genotoxic carcinogens), *p*-NP (genotoxic *in vitro* but not *in vivo* and non-carcinogenic) and CHN (non-carcinogenic and non-genotoxic) (Table II). For the first set of coded compounds (MMS, ENU and CHN), suitable non-cytotoxic starting dose ranges were provided by Covance Laboratories, whereas the individual laboratories had to determine the appropriate dose range for the remaining compounds (2,4-DAT and *p*-NP).

### Controls

Untreated, solvent (acetone) and positive controls [5 µg/cm^2^ (Henkel and TNO) or 16 µg/cm^2^ (P&G) MMS] were run simultaneously with the coded chemicals in each experiment. Mean % tail DNA values of the untreated, solvent and positive controls run in each laboratory are shown in Table III. All experiments reported here were considered valid. Within each laboratory, the % tail DNA of the untreated control was comparable to the solvent control.

**Table III.** Percentage tail DNA of solvent and positive controls at Henkel, P&G and TNO (mean of experiments ± SD) and number of experiments (*n*)

	Untreated control	Solvent control (acetone)	Positive control (MMS)	*n*
Henkel	8.8±6.3	8.1±6.3	35.9±7.5	11
P&G	21.3±10.0	21.6±9.4	69.9±9.3	8
TNO	17.9±9.0	17.8±8.3	50.4±6.2	8

### Methyl methane sulfonate

**Fig. 4.**
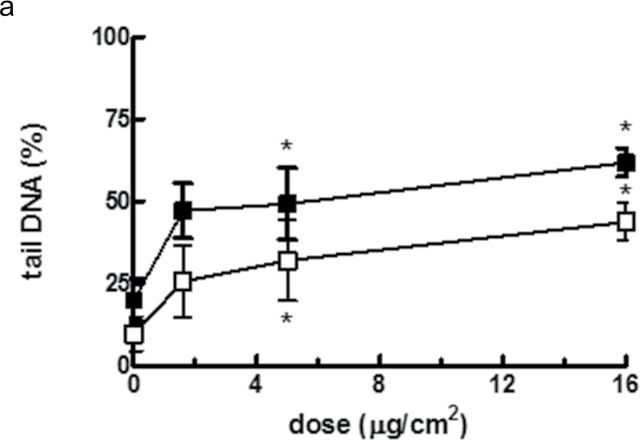

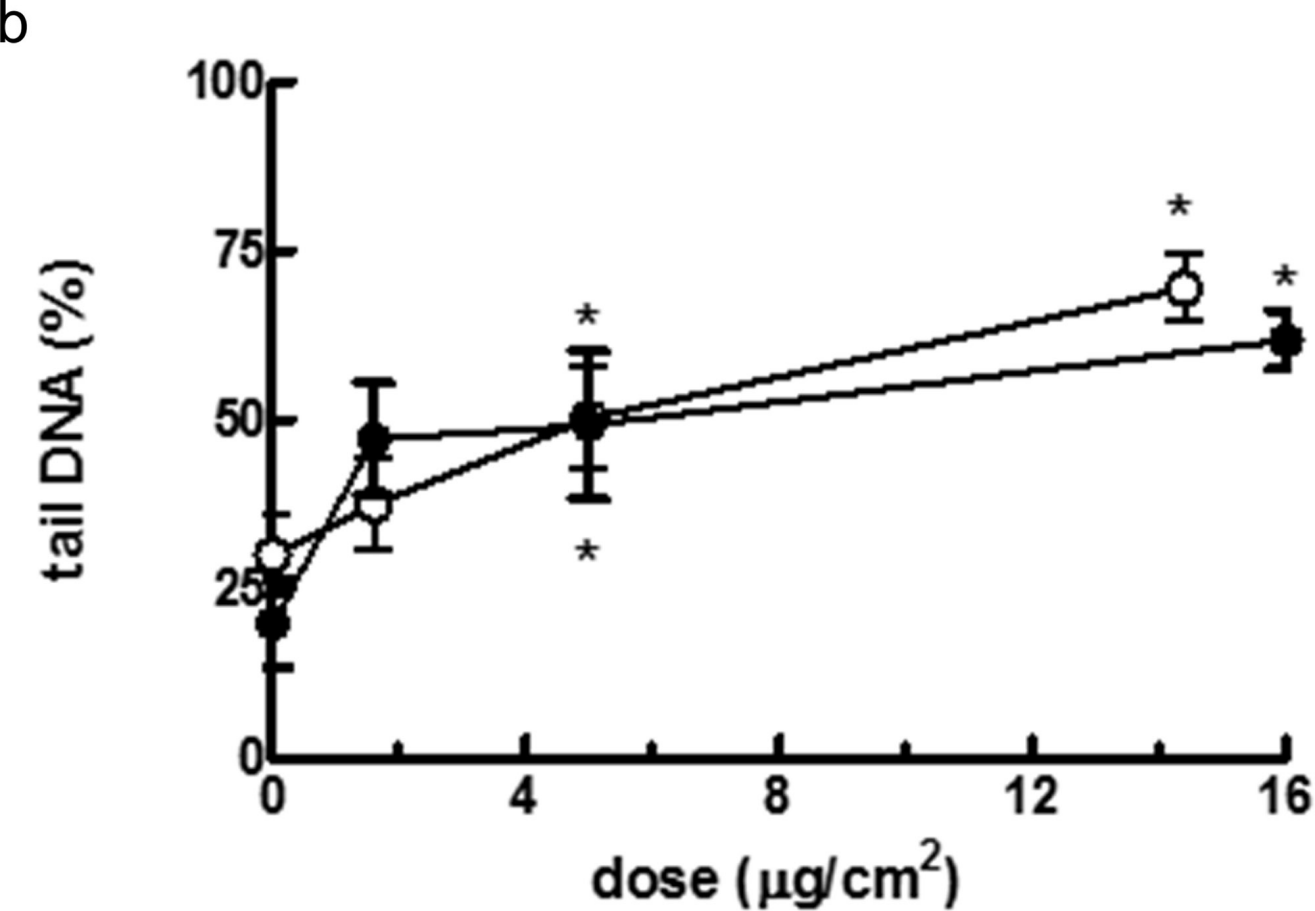

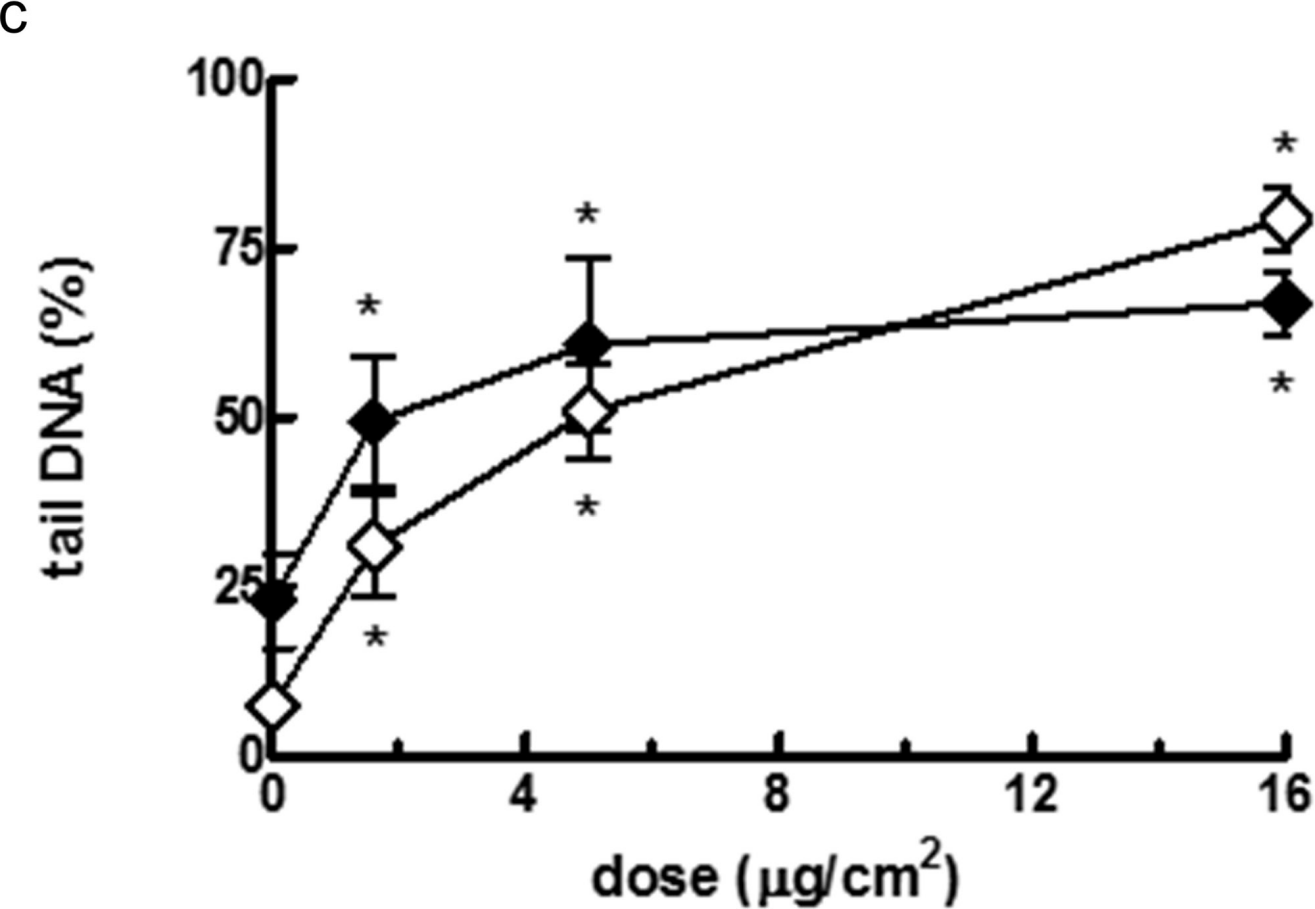
Percentage tail DNA in epidermal cells of EpiDerm™ after 3-h treatment with MMS at (**a**) Henkel, (**b**) P&G and (**c**) TNO. Within each set of results, separate studies are represented with different symbol shapes. Values are mean ± SD. *Significant increase over concurrent solvent control (*P* < 0.05).

A dose-related and statistically significant increase in % tail DNA was observed in tissues treated with MMS in each laboratory and in each experiment (Figure 4). The increase in % tail DNA was statistically significant in all laboratories at two or more dose levels. The maximum observed increase in % tail DNA compared to the solvent control was 10-fold at TNO, up to 4.5-fold at Henkel and up to 2.3-fold at P&G. Although different fold changes were observed across laboratories, statistically significant and reproducible positive responses were observed in all laboratories. The positive responses observed in this pre-validation phase confirmed the results obtained in the earlier harmonisation phase of the project such that all laboratories were able to reproducibly detect a dose-dependent increase in % tail DNA in response to treatment with MMS.

### *N*-Ethyl-*N*-nitrosourea

**Fig. 5.**
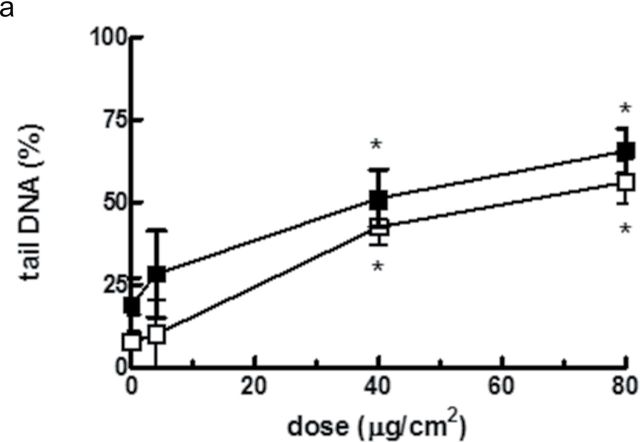

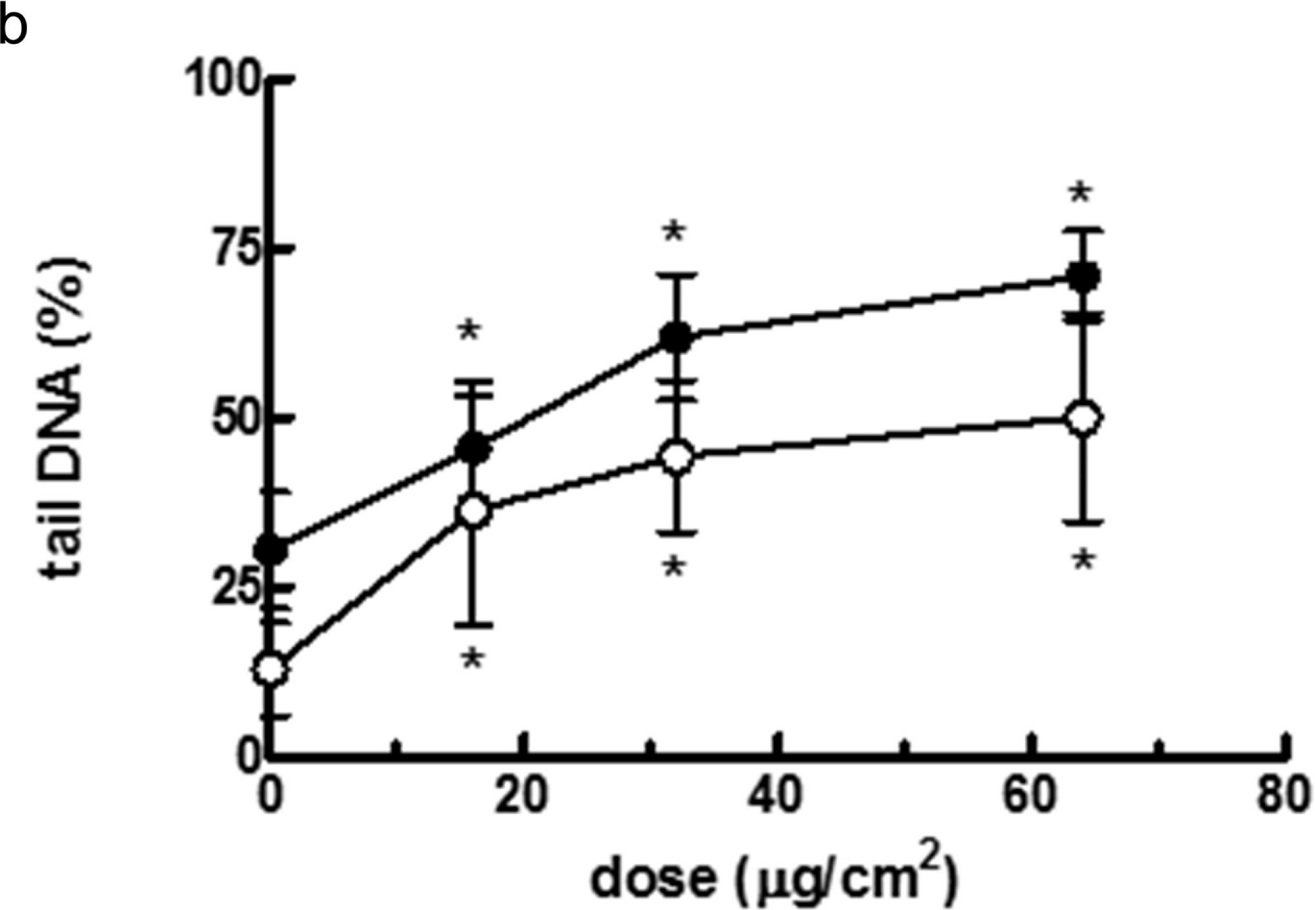

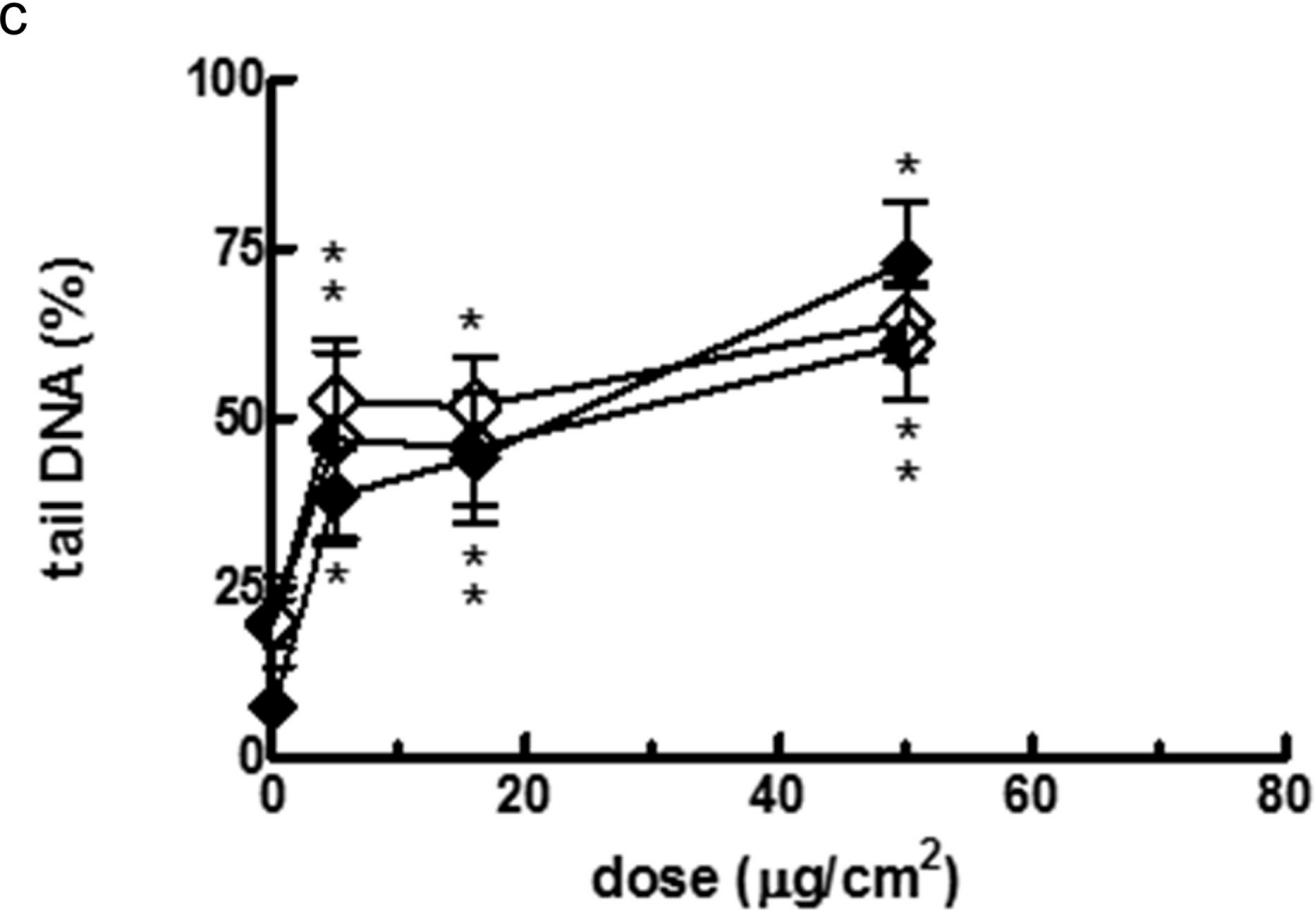
Percentage tail DNA in epidermal cells of EpiDerm™ after 3-h treatment with ENU at (**a**) Henkel, (**b**) P&G and (**c**) TNO. Within each set of results, separate studies are represented with different symbol shapes. Values are mean ± SD. *Significant increase over concurrent solvent control (*P* < 0.05).

ENU resulted in a dose-related increase in % tail DNA in each laboratory and in each experiment (Figure 5). Maximum dose levels tested were 80 µg/cm^2^ (Henkel), 64 µg/cm^2^ (P&G) and 50 µg/cm^2^ (TNO) and caused a maximum increase in % tail DNA compared to the solvent control of 7.3-fold, 3.9-fold and 9.8-fold, respectively. The increase in % tail DNA was statistically significant for at least two consecutive dose levels tested in all laboratories. These results are in line with the findings in the 3D skin micronucleus test and the comet assay performed with *ex vivo* human skin (24,36).

### Cyclohexanone

**Fig. 6.**
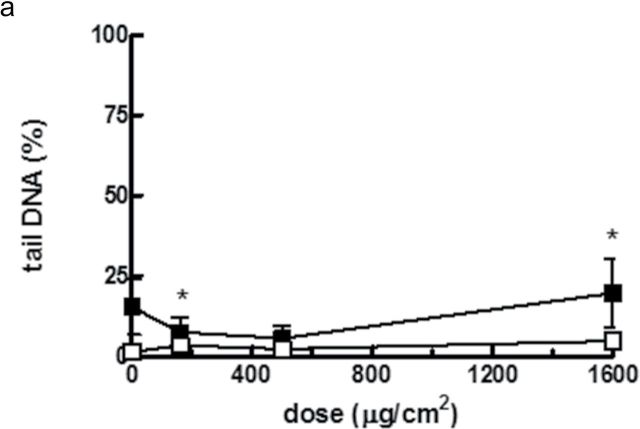

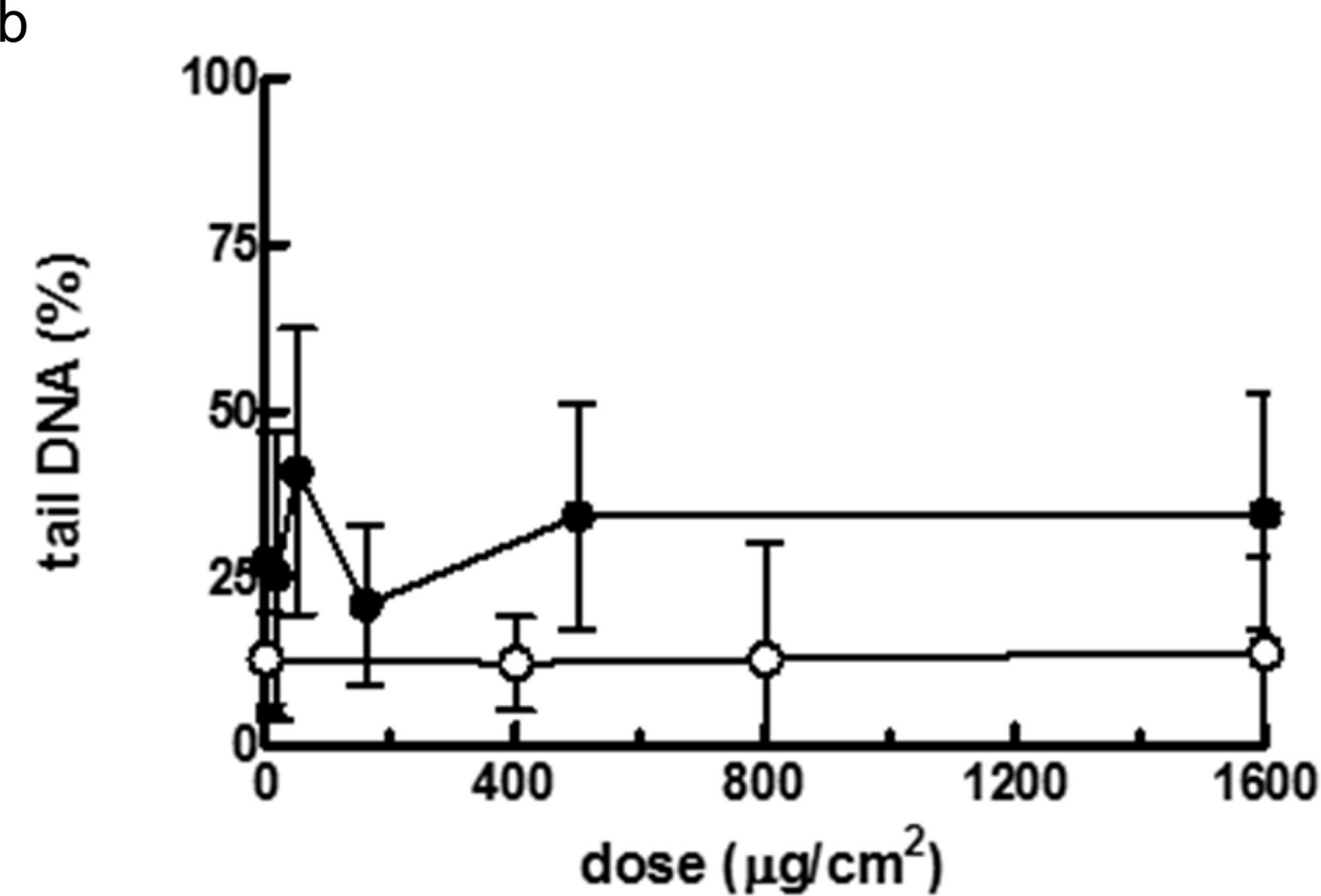

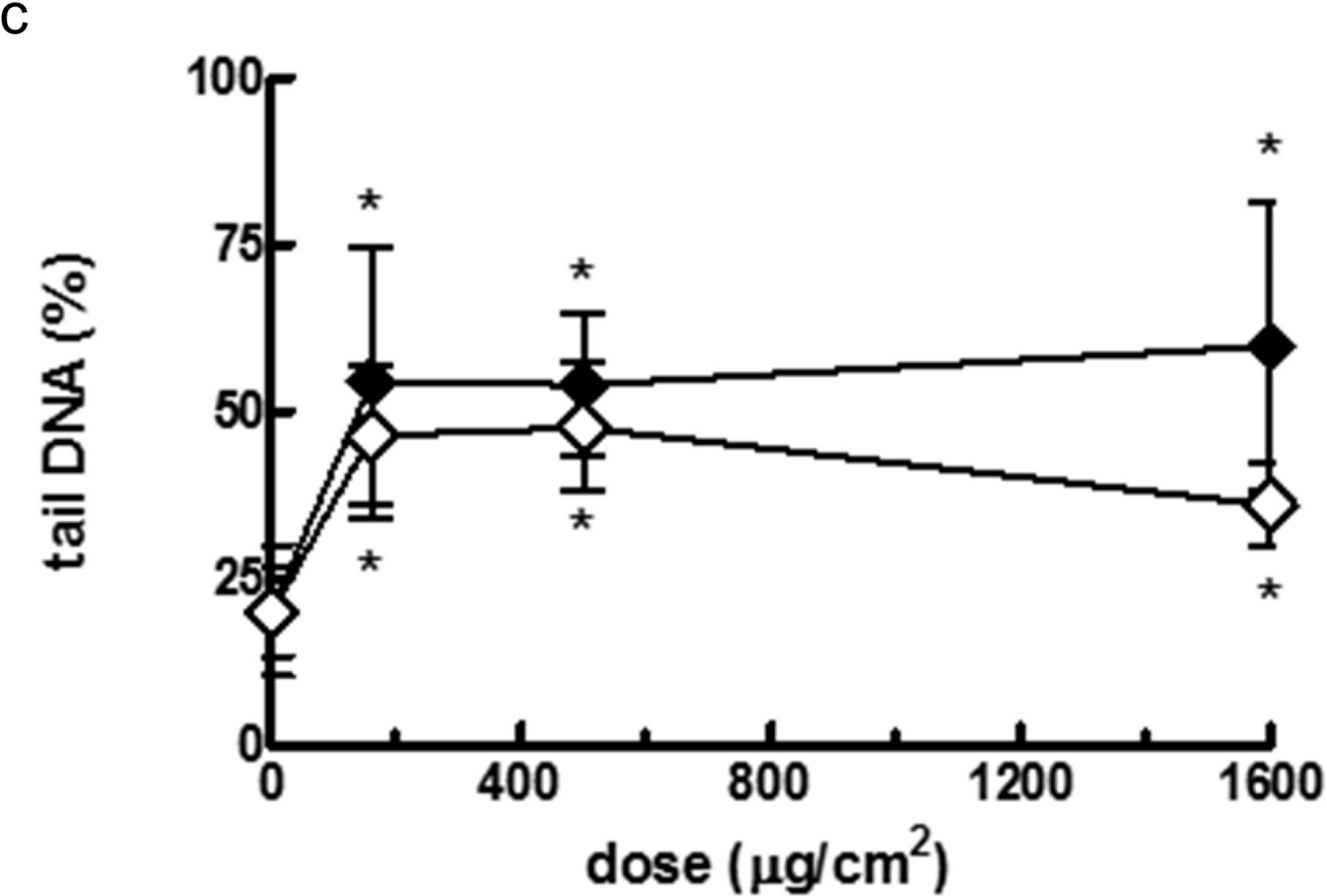
Percentage tail DNA in epidermal cells of EpiDerm™ after 3-h treatment with CHN at (**a**) Henkel, (**b**) P&G and (**c**) TNO. Within each set of results, separate studies are represented with different symbol shapes. Values are mean ± SD. *Significant increase over concurrent solvent control (*P* < 0.05).

CHN was tested up to a maximum concentration of 1600 µg/cm^2^. An increase in % tail DNA compared to the concurrent solvent control was observed at TNO, which was statistically significant at all dose levels tested, but not dose related (maximum increase in % tail DNA compared to the solvent control was 3-fold at 1600 µg/cm^2^) (Figure 6). At Henkel, CHN reported a statistically significant increase at 1600 µg/cm^2^ in one study; however, the % tail DNA values observed in this experiment were relatively low (group means % tail DNA of tissues treated with CHN ranged from 2–5% vs. 1% in the solvent control) (Figure 6a). Therefore, the observed response was considered not biologically relevant. Since the genotoxic response observed by TNO was not dose related and not reproducible across the three laboratories, CHN was considered to be negative overall. Likewise, in the EpiDerm™ micronucleus assay, small but statistically significant increases in micronuclei frequencies were reported in one out of three laboratories but overall, CHN was considered negative due to a lack of dose-related and reproducible increase. By contrast, in the other two laboratories, CHN was clearly negative; therefore, these results confirmed the results obtained in the *ex vivo* human skin comet assay and micronucleus test in EpiDerm™ (24,36).

### 2,4-Diaminotoluene

**Fig. 7.**
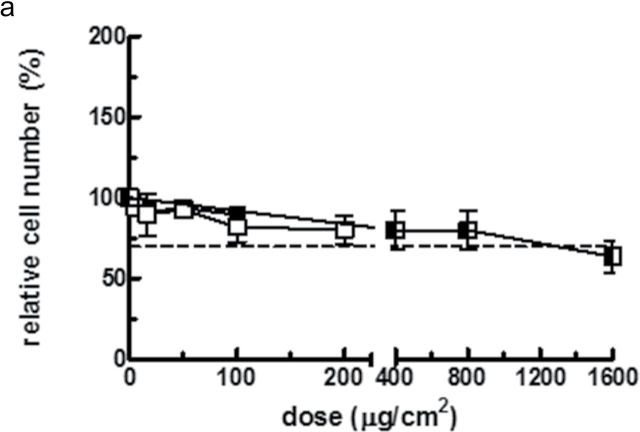

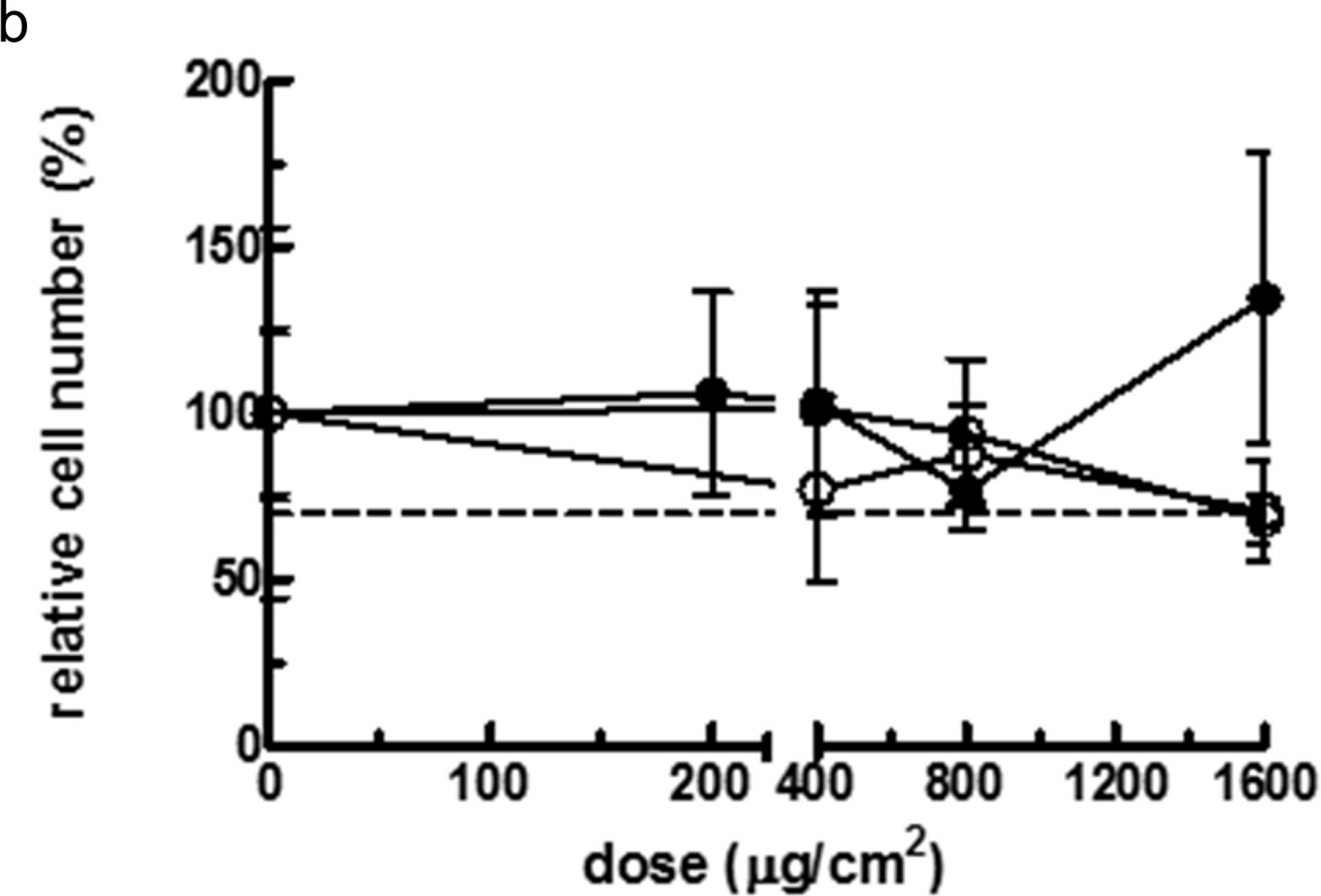

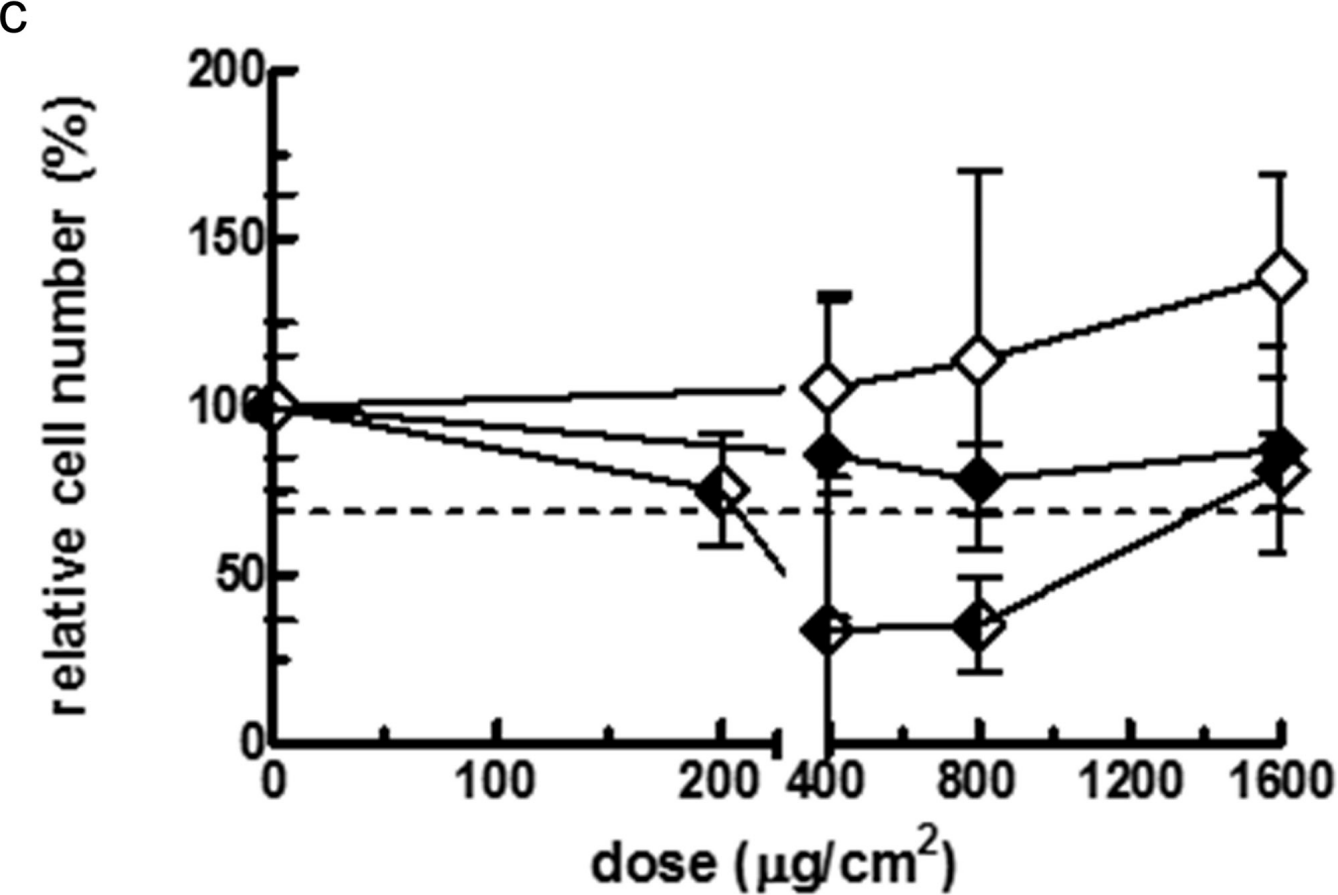
Relative epidermal cell numbers (%) of EpiDerm™ after 3-h treatment with 2,4-DAT at (**a**) Henkel, (**b**) P&G and (**c**) TNO. Within each set of results, separate studies are represented with different symbol shapes. Values are mean ± SD. The dashed line represents the 70% reference line.

**Fig. 8.**
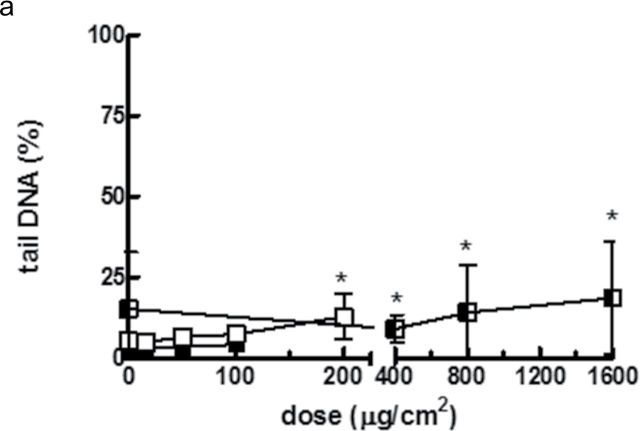

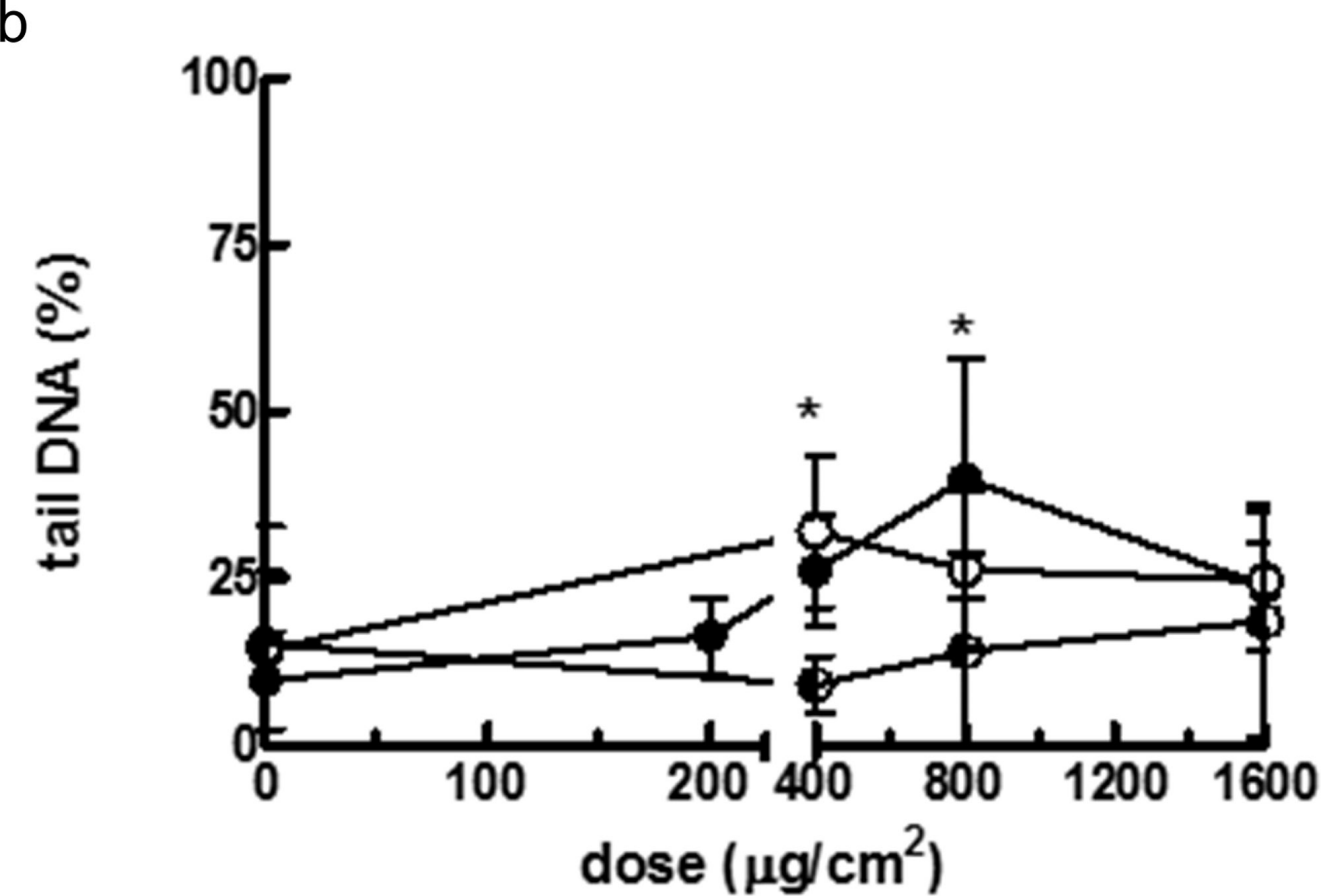

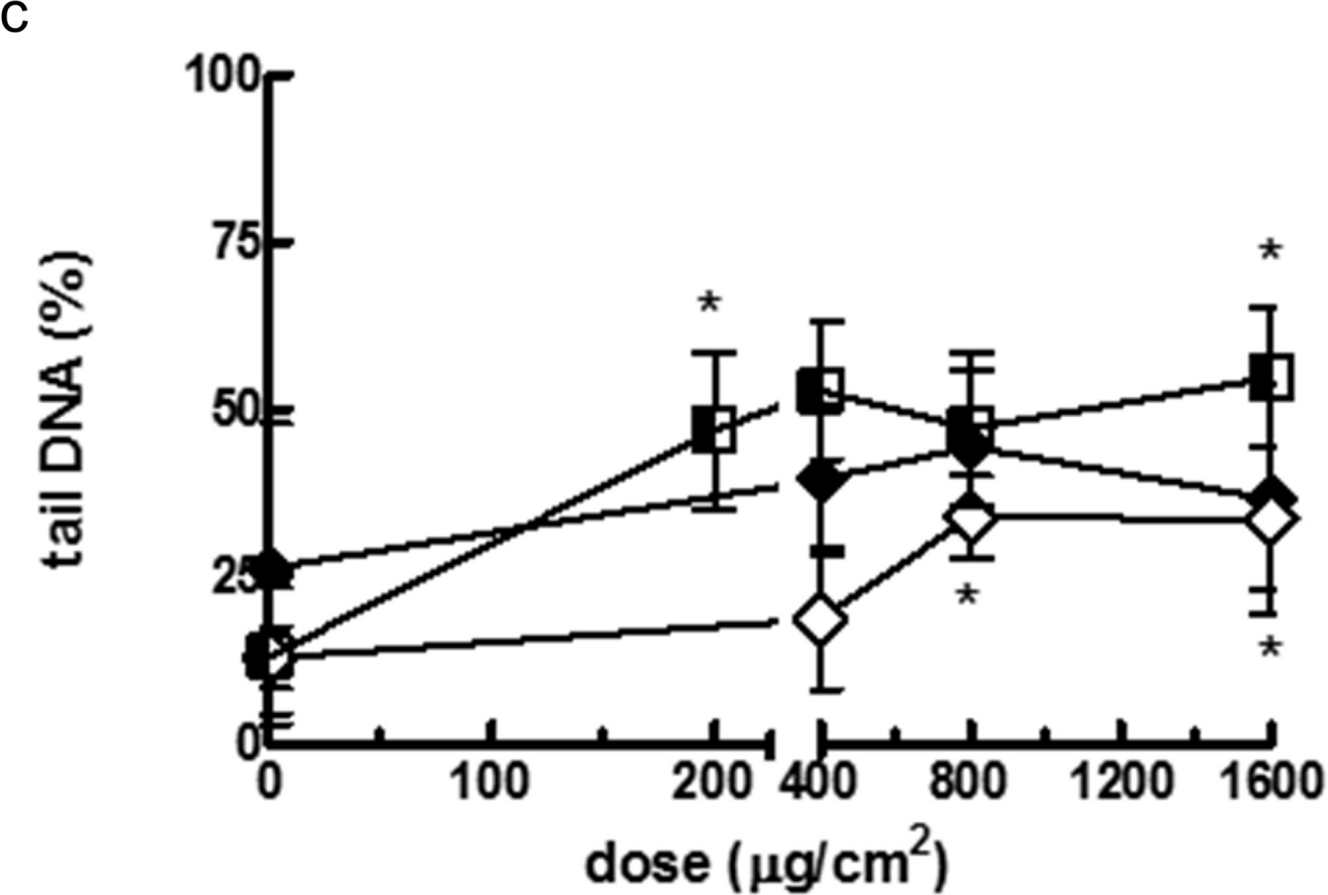
Percentage tail DNA in epidermal cells of EpiDerm™ after 3-h treatment with 2,4-DAT at (**a**) Henkel, (**b**) P&G and (**c**) TNO. Within each set of results, separate studies are represented with different symbol shapes. Values are mean ± SD. *Significant increase over concurrent solvent control (*P* < 0.05).

2,4-DAT resulted in an increase in % tail DNA at acceptable cytotoxicity levels (<30% cytotoxicity) (Figure 7) but was not clearly dose related in all of the experiments (Figure 8). At TNO, the increase in % tail DNA was statistically significant in two studies at two or more dose levels, with a maximum increase of 4.2-fold compared to the solvent control at 1600 µg/cm^2^. At P&G, a statistical increase in % tail DNA was observed in one study at two dose levels (maximum increase was 4.1-fold at 800 µg/cm^2^) and in another study at one dose level. The latter study was considered negative since, according to the evaluation criteria, only the lowest concentration was statistically significant (i.e. there was no trend demonstrated). At Henkel, the increase in % tail DNA was statistically significant in two studies, either at the highest concentration or at all dose levels tested (maximum increase was 8.8-fold at 800 µg/cm^2^). Although each laboratory reported one study that resulted in an increase in % tail DNA that was not statistically significant, the overall response was considered positive because a positive response was observed in two additional experiments. Neither *ex vivo* skin nor EpiDerm™ data was published for 2,4-DAT, but the results of the EpiDerm™ comet assay are in line with *in vivo* genotoxicity and carcinogenicity data (29). 2,4-DAT is a pro-mutagen activated by CYP1A1 in the liver (41) and forms DNA adducts *in vitro* and *in vivo* (42,43). Although expression of CYP1A1 has been reported in EpiDerm™ (9,10), the increase in % tail DNA after treatment with 2,4-DAT was less pronounced compared to the direct-acting compounds MMS and ENU. A less pronounced response of pro-mutagens was also observed in *ex vivo* human skin with benzo[a]pyrene, dimethylbenzantracene and dimethylnitrosamine in the comet assay after a 24-h treatment (36). In the *in vivo* comet assay, a short treatment period of 4h and an extended treatment period of 24h are recommended following treatment (44,45), to detect both rapidly absorbed and direct-acting compounds, and compounds that require time to be absorbed or metabolised. However, 2,4-DAT resulted in a positive response even after a 3-h treatment period and might cause a more pronounced response after longer treatment. The treatment protocol for compounds that require metabolic activation may therefore need further optimisation, e.g. using a multiple treatment protocol and extended treatment periods.

### *p*-Nitrophenol

**Fig. 9.**
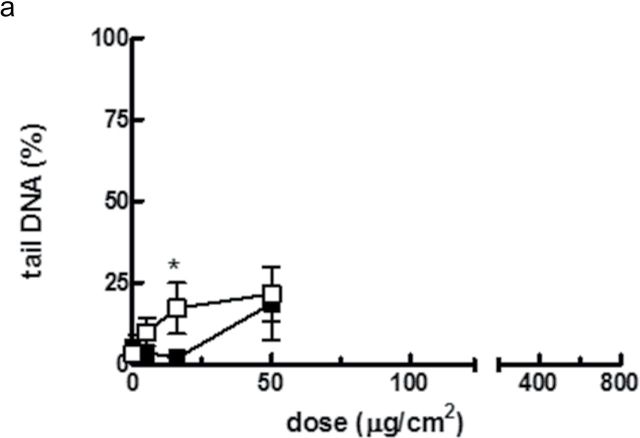

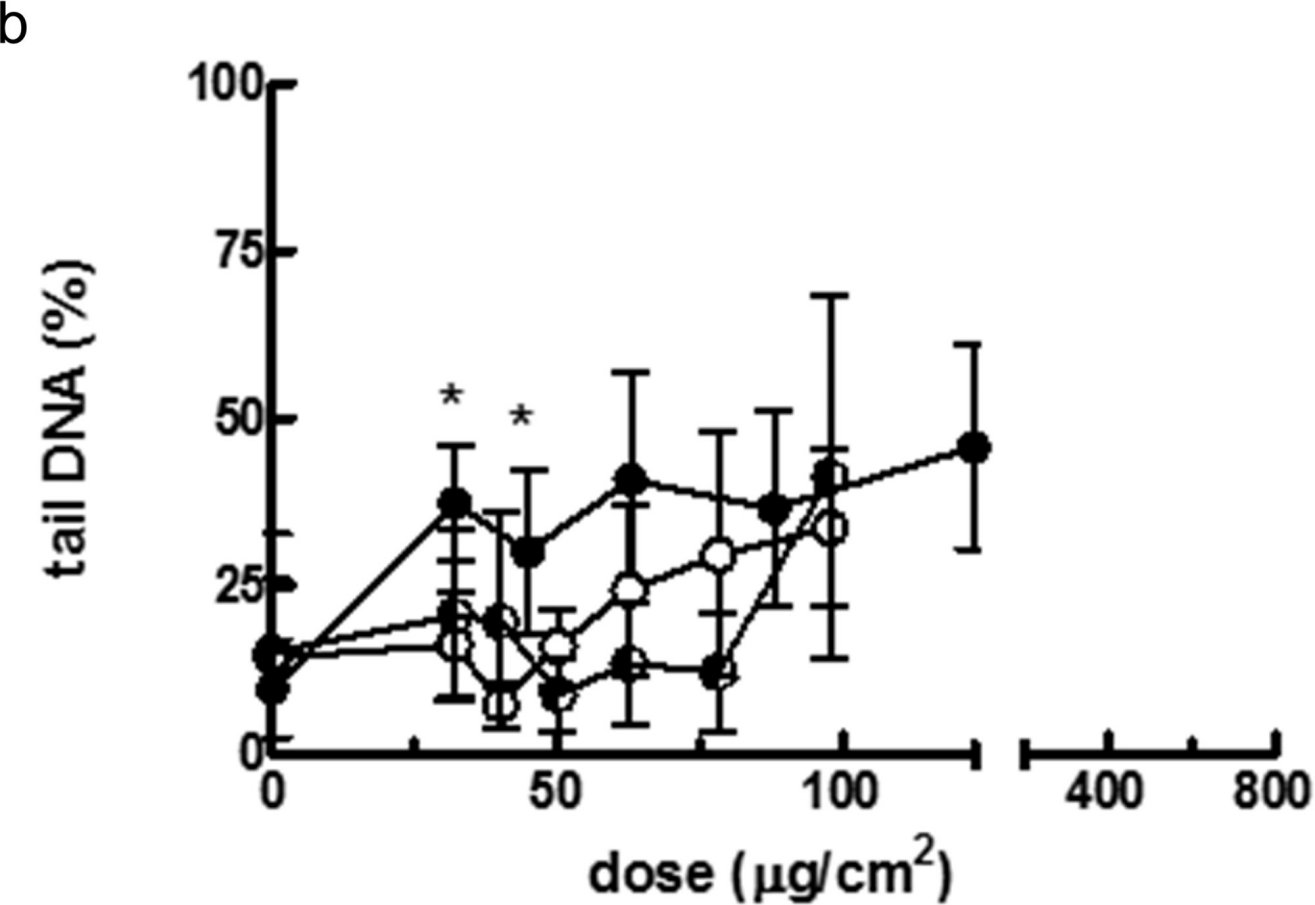

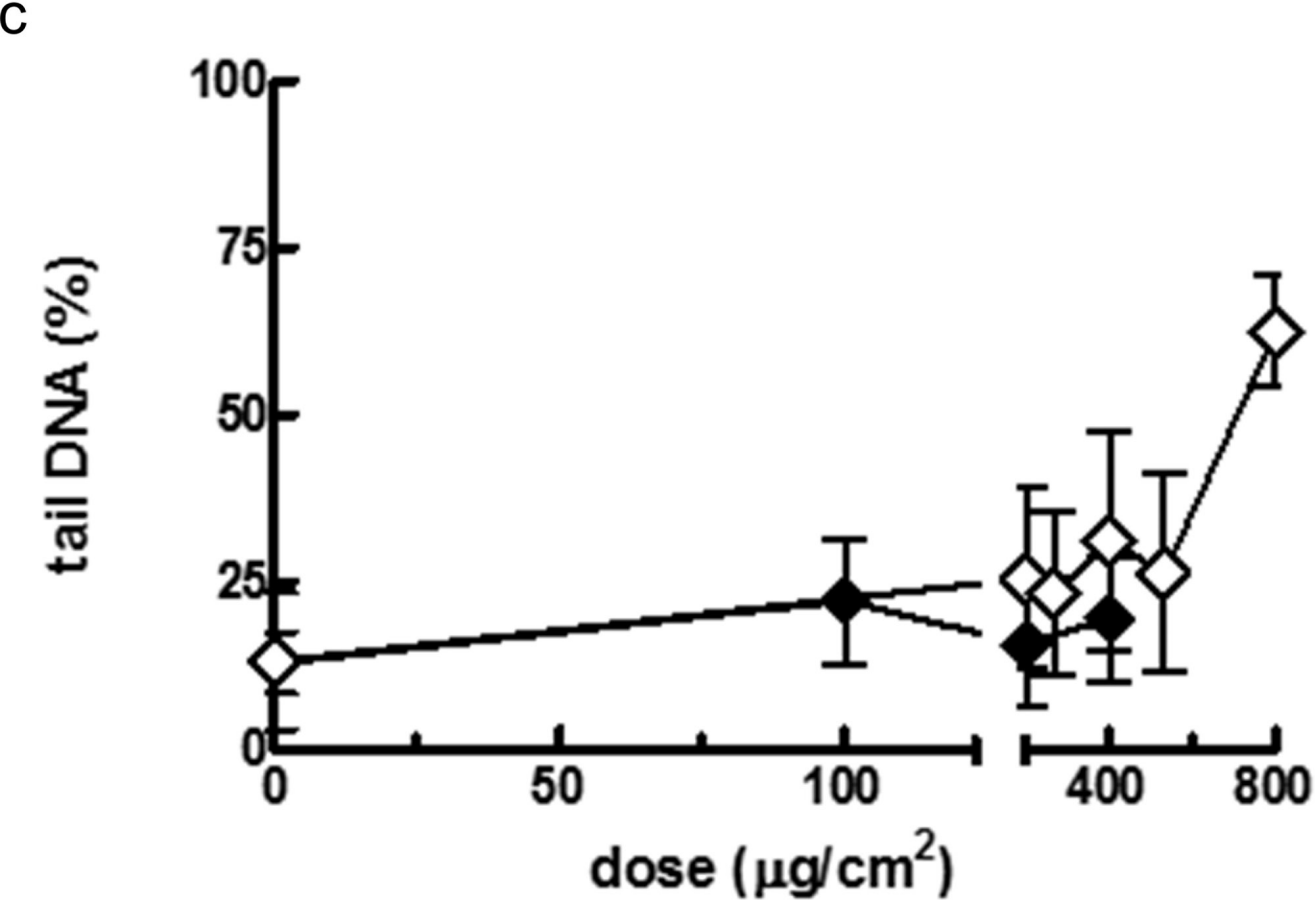
Percentage tail DNA in epidermal cells of EpiDerm™ after 3-h treatment with *p*-NP at (**a**) Henkel, (**b**) P&G and (**c**) TNO. Within each set of results, separate studies are represented with different symbol shapes. Values are mean ± SD. *Significant increase over concurrent solvent control (*P* < 0.05).

**Fig. 10.**
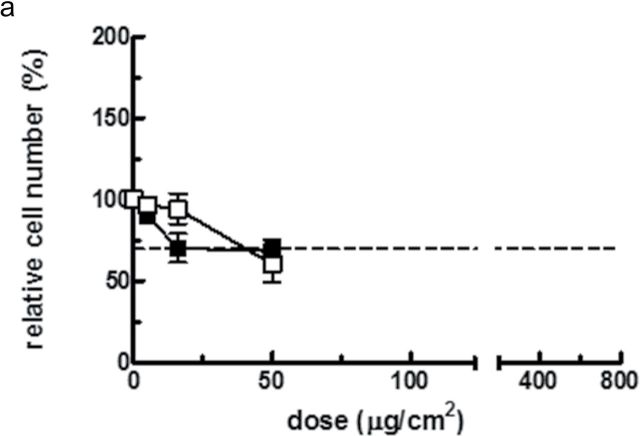

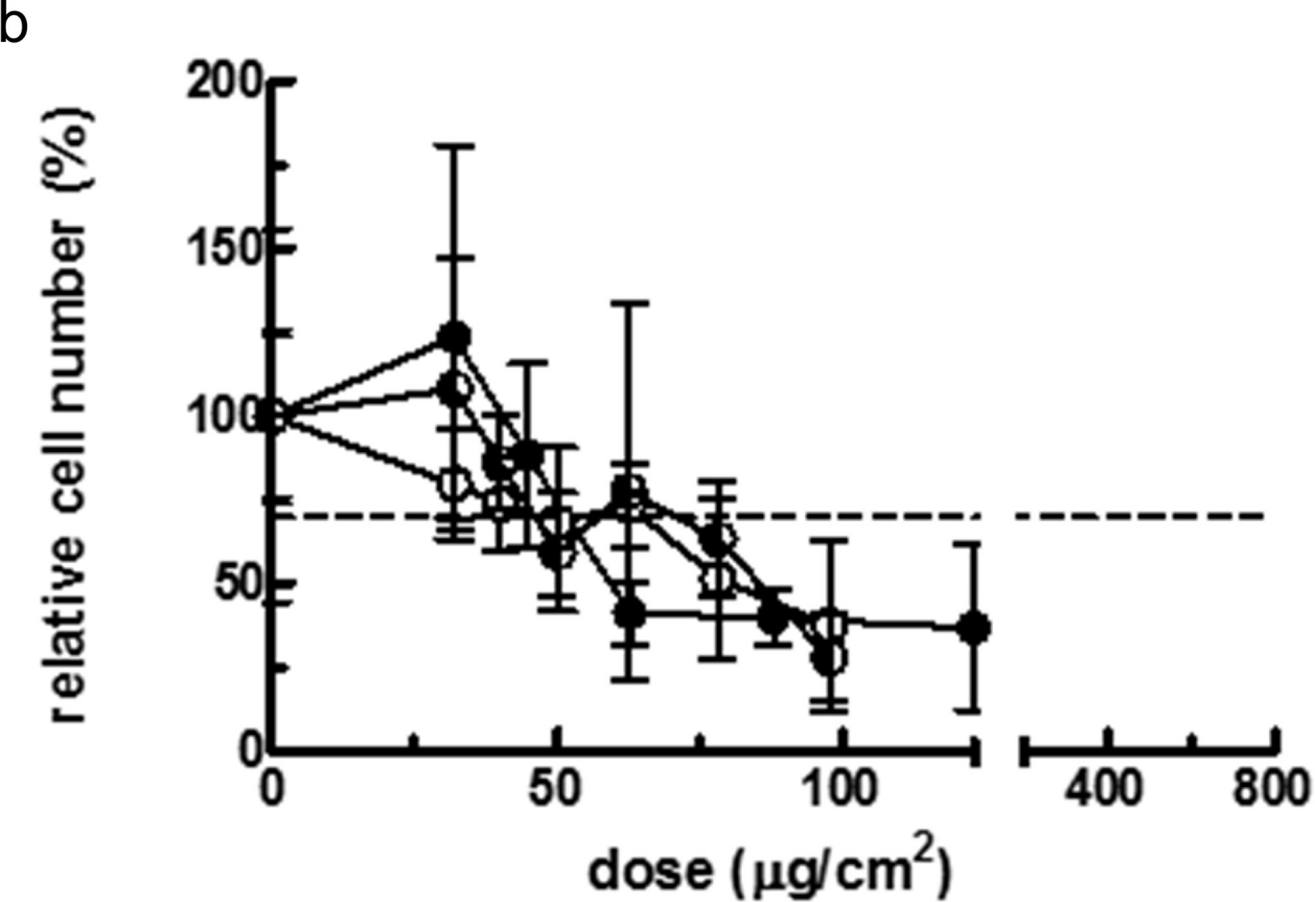

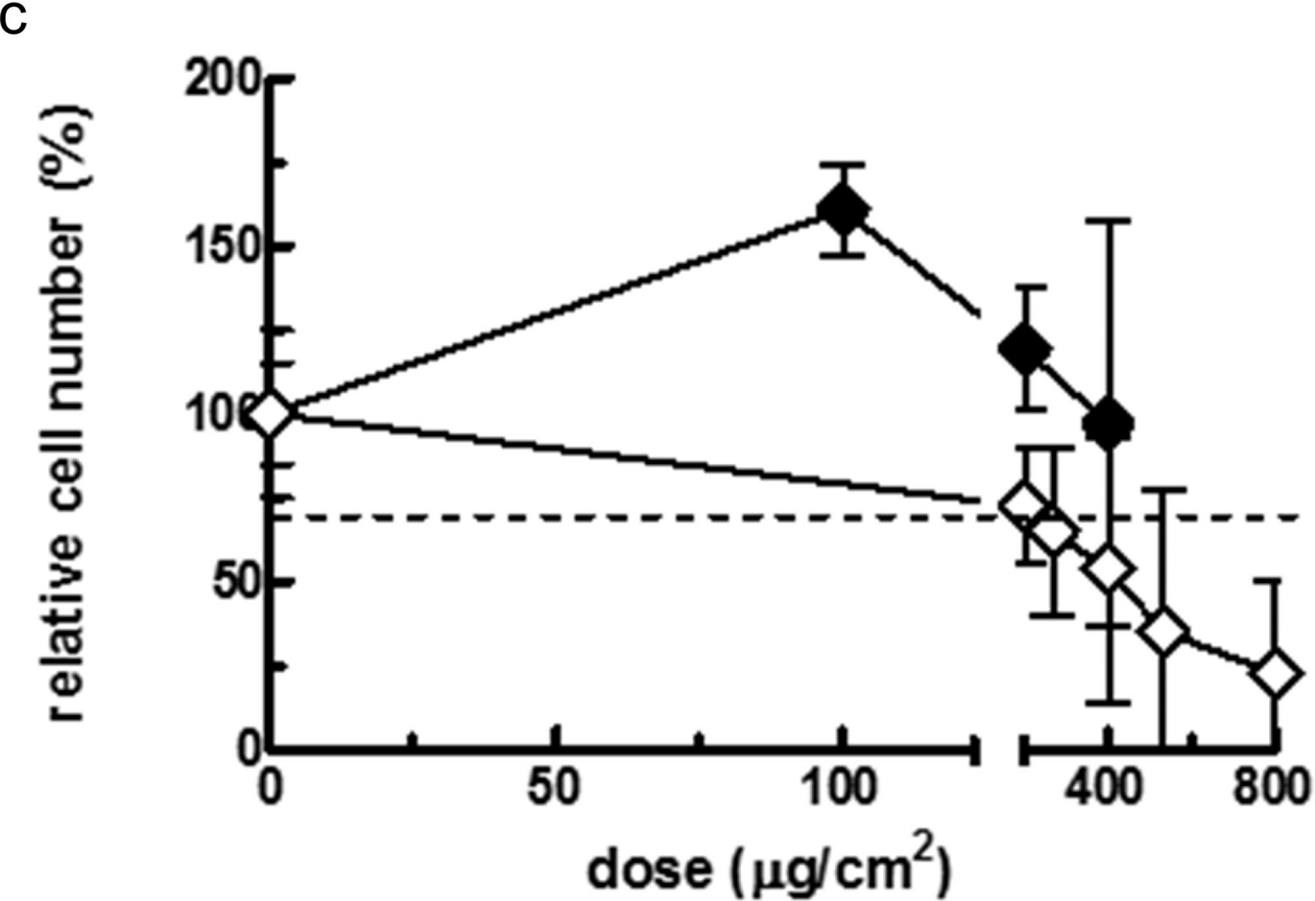
Relative epidermal cell numbers (%) of EpiDerm™ after 3-h treatment with *p*-NP at (**a**) Henkel, (**b**) P&G and (**c**) TNO. Within each set of results, separate studies are represented with different symbol shapes. Values are mean ± SD. The dashed line represents the 70% reference line.

*p*-NP was the only compound showing clear cytotoxic effects within the tested concentration range (Figure 10). At TNO, higher dose levels were tested than at P&G and Henkel, and maximum dose levels were 800 µg/cm^2^, 98 µg/cm^2^ and 50 µg/cm^2^, respectively. The reason for this difference in dose level is unknown. Statistically significant DNA damage occurred only at doses that showed substantial cytotoxicity (>30% cell loss), and the overall response pattern was comparable despite some differences in doses tested (Figures 9 and 10). Henkel results were considered inconclusive because both studies performed did not fulfil the acceptability criteria since there were no sufficient non-cytotoxic doses in order to make a complete analysis. The results, however, fit the pattern observed by the other laboratories. Overall, *p*-NP was considered negative in the 3D skin comet assay because the positive responses observed in the 3D skin comet assay were associated with excessive cytotoxicity. Similar results were observed in studies using *ex vivo* human skin (36). Excessive cytotoxicity is one of the reasons for the unacceptably high false positive rate in current regulatory *in vitro* genotoxicity assays (3). *p*-NP was indeed listed as a misleading positive that was reported to be genotoxic *in vitro*, but was non-carcinogenic *in vivo* (29).

### Reproducibility

High (>30%) and variable % tail DNA values of untreated and solvent control tissues (negative controls) were periodically observed in all three laboratories, but not always in the same batches (data not shown). Generally, higher % tail DNA values in negative controls were associated with a greater variability than those with lower values. Several attempts were made to investigate the reason for the high and variable % tail DNA in the negative controls. The use of underdeveloped skin models (EPI-201, MatTek Corporation) was investigated in one of the participating laboratories to address any possible stress-related issues that may occur during shipment of the tissues. The underdeveloped tissues were shipped 4 days earlier and then cultured further at the laboratory in which the test was performed. This generally resulted in a reduction of both the % tail DNA values and variability of untreated and solvent controls, whereas the positive control still resulted in % tail DNA values that were comparable to normal EpiDerm™ (EPI-200) skin models from the same batch (data not shown). These limited data suggest that the use of underdeveloped tissues could help reduce variability. Not all the participating labs, however, were able to use EPI-201 for logistical reasons (arrival of tissues on the weekend if shipped from the USA to Europe).

To investigate epidermal donor variability, MMS was tested on EpiDerm™ tissues produced with keratinocytes from three different donors, namely, donors 1188, 254 and 219 (Table IV). Marginally lower % tail DNA in the untreated and solvent control was observed in tissues from donor 219 compared to donors 254 and 1188; however, these results were based on a single experiment for donor 219. Overall, the donor variability was within an acceptable range and the skin models reconstructed from epidermal cells from all investigated donors responded similarly to MMS and were considered suitable for the 3D skin comet assay. Also, when comparing two different donors within the same experiments (i.e. simultaneous treatment, cell isolation and electrophoresis), the results were highly comparable. As the results are based on a limited number of experiments and donors, further investigation may be warranted to determine donor variability in more detail.

**Table IV.** Percentage tail DNA after exposure to MMS in donors 219, 254 and 1188 (mean of experiments ± SD) and number of experiments (*n*)

	219	254	1188
Untreated control	14.6	14.8±9.8	22.2±6.7
Solvent control (acetone)	13.6	27.0±7.8	27.3±8.2
1.6 µg/cm^2^ MMS	40.1	41.1±4.7	46.1±9.6
5 µg/cm^2^ MMS	53.0	55.4±6.6	61.3±15.2
16 µg/cm^2^ MMS	70.6	62.9±18.8	69.2±12.9
*n*	1	3	4

Within the laboratories, the results were reproducible, e.g. both experiments of each laboratory showed a comparable response. Some inter-laboratory variability was observed for CHN (which was only reproducibly positive at TNO) and *p*-NP (for which there were large differences in the dose levels tested and an inconclusive result was observed at Henkel), but not for the true positive compounds, MMS, 4NQO, ENU and 2,4-DAT. Generally, the % tail DNA values measured at Henkel were lower both for solvent and positive controls compared to P&G and TNO.

### Cytotoxicity

The assessment of cytotoxicity in parallel to genotoxicity is extremely important in order to exclude a positive genotoxic response caused by excessive cytotoxicity. Moreover, cytotoxicity evaluation is helpful to select suitable dose levels for genotoxicity studies. The applicability of several cytotoxicity parameters was investigated, including trypan blue dye exclusion, ethidium bromide/acridine orange staining, cell counts and the MTT test. The MTT test was used previously in combination with the % ghost cells in *ex vivo* human skin to exclude the possibility that a positive response observed in the comet assay was due to excessive cytotoxicity. After 3-h treatment, cytotoxicity measured using the MTT test did not correlate with the appearance of the comets on the slides, probably because incubations longer than 3h are necessary to detect a cytotoxic effect using this measurement. Therefore, the treatment period for the MTT test was extended to 24h in *ex vivo* human skin tissues (36). Similar results with respect to exposure time and correlation with cytotoxicity were obtained with EpiDerm™ (data not shown). The disadvantage of the MTT test, however, is that it cannot be performed in the same skin model as used for the comet assay. Simultaneous cytotoxicity assessment and comet evaluation in the same skin model are preferred for ultimate comparison and save time and costs. Trypan blue dye exclusion and ethidium bromide/acridine orange staining were also tested but were considered not to be sufficiently sensitive. Additional ongoing investigations include the feasibility of using the measurement of cellular ATP levels as a parameter for cytotoxicity. In this case, as for MTT, a protocol extending beyond 3-h treatment will be helpful. In the meantime, however, relative cell counts proved to be a good alternative for sensitive detection of cytotoxicity in 3D skin tissues.

## Conclusion

This paper describes the development and optimisation of a comet assay protocol for EpiDerm™ skin models, as well as intra- and inter-laboratory reproducibility results from the testing of five coded compounds. The EpiDerm™ comet assay methodology was readily adapted and harmonised between the laboratories. Negative and positive controls were reproducible within and across the laboratories. There was a good intra- and inter-laboratory reproducibility since all three genotoxic carcinogens were correctly identified in all three laboratories. Both non-carcinogens were also negative in the EpiDerm™ comet assay in all but one laboratory (one false positive result). These results support the conclusion that the comet assay in EpiDerm™ skin models is a promising model for genotoxicity testing of compounds with a dermal route of exposure. The work presented here will provide a starting point for further research. The predictive capacity of the comet assay in reconstructed skin models will be established further and involves more laboratories and other skin model types, including reconstructed full-thickness skin models.

## Funding

This work was supported by Cosmetics Europe and the European Union Reference Laboratory on Alternatives to Animal Testing (contract no. CCR.IHCP.C432901.X0, CCR.IHCP.C435135.X0).
